# A Pepper *MSRB2* Gene Confers Drought Tolerance in Rice through the Protection of Chloroplast-Targeted Genes

**DOI:** 10.1371/journal.pone.0090588

**Published:** 2014-03-10

**Authors:** Joung Sug Kim, Hyang-Mi Park, Songhwa Chae, Tae-Ho Lee, Duk-Ju Hwang, Sung-Dug Oh, Jong-Sug Park, Dae-Geun Song, Cheol-Ho Pan, Doil Choi, Yul-Ho Kim, Baek Hie Nahm, Yeon-Ki Kim

**Affiliations:** 1 Division of Bioscience and Bioinformatics, Myong Ji University, Yongin, Kyonggido, Korea; 2 Genomics Genetics Institute, GreenGene BioTech Inc., Yongin, Kyonggido, Korea; 3 Rice research division, National Institute of Crop Science, Suwon, Korea; 4 National Academy of Agricultural Science, Rural Development Administration, Suwon, Korea; 5 College of Agriculture and Life Sciences and Plant Genomics & Breeding Institute, Seoul National University, Seoul, Korea; 6 Functional Food Center, Korea Institute of Science and Technology (KIST), Gangneung, Gangwon-do, Korea; University of Delhi South Campus, India

## Abstract

**Background:**

The perturbation of the steady state of reactive oxygen species (ROS) due to biotic and abiotic stresses in a plant could lead to protein denaturation through the modification of amino acid residues, including the oxidation of methionine residues. Methionine sulfoxide reductases (MSRs) catalyze the reduction of methionine sulfoxide back to the methionine residue. To assess the role of this enzyme, we generated transgenic rice using a pepper *CaMSRB2* gene under the control of the rice *Rab21* (responsive to ABA protein 21) promoter with/without a selection marker, the bar gene.

**Results:**

A drought resistance test on transgenic plants showed that *CaMSRB2* confers drought tolerance to rice, as evidenced by less oxidative stress symptoms and a strengthened PSII quantum yield under stress conditions, and increased survival rate and chlorophyll index after the re-watering. The results from immunoblotting using a methionine sulfoxide antibody and nano-LC-MS/MS spectrometry suggest that porphobilinogen deaminase (PBGD), which is involved in chlorophyll synthesis, is a putative target of *CaMSRB2*. The oxidized methionine content of PBGD expressed in *E. coli* increased in the presence of H_2_O_2_, and the Met-95 and Met-227 residues of PBGD were reduced by *CaMSRB2* in the presence of dithiothreitol (DTT). An expression profiling analysis of the overexpression lines also suggested that photosystems are less severely affected by drought stress.

**Conclusions:**

Our results indicate that *CaMSRB2* might play an important functional role in chloroplasts for conferring drought stress tolerance in rice.

## Introduction

Plants are exposed to various harsh environmental stresses including light, drought, salinity, high temperatures, and pathogen infections. Among these, drought is becoming an increasingly severe problem in many regions with increasing population and global climate change and it is one of the major limiting factors for crop production and quality [1]–[3]. To overcome drought stress, transgenic crop approaches have been suggested. For example many of the key enzymes involved in ABA synthesis and signaling/regulatory pathways have been investigated transgenically in relation to improving plant drought tolerance [4], [5].

One of the unavoidable consequences of drought stress is the production of reactive oxygen species (ROS) in mitochondria, chloroplasts, and peroxisomes [2]. These species include singlet oxygen (^1^O_2_), superoxide radical (O_2_^−^), hydrogen peroxide (H_2_O_2_), and hydroxyl radical (HO^·^). Some ROS are highly toxic so they are rapidly detoxified by enzymatic and non-enzymatic scavenging systems [6]. There are many attempts to improve stress tolerance in plants by modifying expression of several ROS-scavenging enzymes [7]. For example, the expression of manganese superoxide dismutase (MnSOD) from pea under the control of an oxidative stress-inducible *SWPA*2 promoter can improve drought tolerance in rice [8]. However, excessive enhanced ROS can cause oxidative damage to multiple cellular components, including proteins, lipids, DNA and RNA. Until now, while mechanisms to repair DNA damage by the ROS have been well-studied [9], [10], the protein repair mechanism has been much less analyzed.

The oxidative modification of amino acids by the ROS can lead to protein conformational changes and the loss of function [11]. Fortunately, some types of protein damage are reversible through the action of cellular repair systems. Methionine (Met), in particular, is highly susceptible to oxidative damage, which converts Met into methionine sulfoxide (MetSO), which can exist in the R or S configuration. MetSO is readily reversed by the methionine sulfoxide reductases A and B (MSRA and MSRB), which are specific for the S and R stereoisomer forms, respectively [12]. MSRA and MSRB do not share identity at the level of their primary sequences nor at the three-dimensional structure level [12]. All plants possess multigene families of MSRA and MSRB. In Arabidopsis, five MSRA and nine MSRB orthologs have been identified, and differential regulation patterns in the chloroplast and cytosol have been reported [13]. Many studies have demonstrated the function of MSRA in plant responses to oxidative stress. However, very little information is available regarding MSRB. T-DNA insertional mutations and the overexpression of *AtMSRB3* result in reduced and enhanced tolerance to oxidative stress, respectively [14]. In a recent report, an Arabidopsis MSRB1 and MSRB2 knockout mutant showed reduced growth in plants cultivated under high light or low temperature conditions [15]. In rice, only four MSRA (OsMSRA2.1, OsMSRA2.2, OsMSRA4, and OsMSRA5) and three MSRB (OsMSRB1, OsMSRB3, and OsMSRB5) genes exist [13]. The overexpression of either OsMSRA4 or OsMSRB1 in yeast showed enhanced cellular resistance to oxidative stress, and OsMSRA4-overexpressing transgenic rice plants also showed enhanced tolerance under salt treatment [16].

A pepper (*Capsicum annuum*) methionine sulfoxide reductase B2 gene (CaMSRB2) has been implicated in the ROS-generating response of hosts against pathogens [17]. The down-regulation of this gene in pepper using virus-induced gene silencing resulted in accelerated cell death from an incompatible bacterial pathogen and the enhanced susceptibility to a compatible bacterial pathogen. In contrast, the *CaMSRB2*-overexpressing transgenic tomato plants showed a reduced production of hydrogen peroxide and exhibited an increased resistance to the pathogens. These data suggest that gene products, via the regulation of their methionine oxidation, are critical in the active defense against pathogens and that *MSRB2* might be implicated in the regulation. These results raise several interesting points, including an imminent question as to which proteins are affected under stress conditions. In addition, the effects of MSRB overexpression may not be solely confined to biotic stress because the ROS are also produced during abiotic stresses, such as drought and cold [2], [6]. Methionine oxidation could be a common protein posttranslational modification, as the ROS production increases under adverse conditions; therefore, MSRB might play a critical role in enzyme recovery by reducing the oxidized proteins.

Few reports have identified the target proteins of the MSRs. In *Arabidopsis thaliana*, MSRs protect the chaperone-like activity of a small heat shock protein [18]. MSRBs account for most of the leaf peptide MSR activity and are essential for the preservation of photosystem antennae under environmental constraints [15]. However, how these enzymes are involved in the modulation of gene expression is unknown. In this regard, a specialized system called retrograde (organelle-to-nucleus) signaling through which the nuclear gene expression is controlled by the organelle status could implicate the functionality of MSRs. As many proteins are imported into the chloroplast and mitochondria, uncoordinated gene expression could be detrimental under stress conditions. It has been shown that the changes in the redox status or energy balance of the organelles, especially the chloroplast, could be conveyed to the nucleus through redox-controlled protein phosphorylation and sensory systems [19]–[21]. For example, ROS accumulate when the plants are exposed to excess light [6]. Among the ROS, H_2_O_2_ functions as a retrograde signal candidate [22], [23]. In addition, a genetic approach using Arabidopsis mutants (designated as genome-uncoupled, gun) suggested that genes that are involved in tetrapyrrole synthesis are related to retrograde signaling and that metabolic imbalance increases the chlorophyll intermediate Mg-protoporphyrin IX (Mg-ProtoIX), which initiates retrograde communication between the chloroplast and the nucleus [24]. It is plausible that MSRs could be directly or indirectly involved by converting oxidized Met and recovering the activity of some of the proteins that are involved in these processes.

Here, we report that *CaMSRB2* might have undergone a subtly different evolutionary path than that of many dicot plants, as evidenced by the analysis of the overexpression of *CaMSRB2* in rice, a monocot model plant [17]. This gene seems to be involved in plant responses to the biotic and abiotic stresses where ROS are generated. The immunoblotting and GC-Mass analysis indicate that porphobilinogen deaminase (*PBGD*) might be a target protein of CaMSRB2. The genome-wide expression profiling also suggests that photosynthesis-related genes in the transgenic lines are less severely down-regulated, implicating the importance of MSRB2 and raising the possibility of its functioning in the retrograde signaling to control gene expression in the nucleus. These data suggest that plant MSRBs play important roles in homeostasis in chlorophyll synthesis during the drought response in part by recovering damaged PBGD.

## Materials and Methods

### Analysis of ortholog groups

Genes in a family were retrieved from the RAP2 rice genome annotation (http://rapdb.dna.affrc.go.jp/), TAIR Arabidopsis genome annotation version 9 (http://www.arabidopsis.org/), and CaEST DB using BLASTp. To draw the unrooted phylogenetic tree, a two-step analysis was applied. First, the ortholog groups were tested with OrthoMCL [25]. This analysis compartmentalized the family into two groups. Second, the amino acid sequences of the group were aligned with ClustalW, and a distance matrix of the alignment was then calculated by the Prodist program in the Phylip package [26]. The matrix was transformed into a tree by the Neighbor program in the Phylip package as well.

### Plasmid construction

The *Rab21* promoter sequence (1.6 kb) was amplified using a promoter-specific primer pairs from the plasmid *Rab21:gfp* [27] as template. The *rbcS* promoter-*Tp* sequence from pSB-RTG [28] was replaced by the *Rab21* promoter, resulting in the plasmid pSB-Rab21. pSB-RTG contains the potato protease inhibitor II terminator/35S promoter/bar/nopaline synthase terminator between the right and left border sequences of pSB11 [28]. A Gateway cassette containing attR recombination sites flanking a ccdB gene and a chloramphenicol-resistance gene are blunt-end cloned into the plasmid pSB-Rab21, which yielded the plasmid pSB-RbGW. A full-length cDNA of *CaMSRB2* that was obtained from Dr. D.I. Choi [17] was inserted into the binary vector pSB-RbGW by an LR reaction following the manufacturer's instructions (Invitrogen), which produced the plasmid *MSRB2-Bar*. Additionally, the *CaMSRB2* gene was cloned into the pSB-Rab21 without the potato protease inhibitor II terminator/35S promoter/bar, resulting in the plasmid *MSRB2*-mini. Finally, the constructs were introduced into *Agrobacterium tumefaciens* LBA4404 by triparental mating [29].

For subcellular localization, the *CaMV 35S* promoter and nos terminator from pSB-RTG were amplified and cloned into pGEM T-Easy vector, resulting in the plasmid pG-P35T. OsMSRB3 and OsMSRB5 were isolated by RT-PCR from four-week-old Oryza sativa cv. IlmiI seedlings using the primers indicated (Table S1). The full-length coding region of these *MSRB* genes was cloned into the pET-DsRed expression vector [30] to generate the *MSRB:DsRed* fusion genes. The DsRed *-*fused genes and *GFP* were individually cloned into the plasmid pG-P35T. The *rbcS* promoter-*Tp* sequence and nos terminator from pSB-RTG were amplified and cloned into pGEM T-Easy vector (Promega, www.promega.com), in which *GFP* was cloned for using a chloroplast target marker.

Porphobilinogen deaminase (PBGD, Os02g0168800), ferredoxin—NADP reductase (Os06g0107700), dihydrodipicolinate reductase 1 (Os02g0436400), ribulose bisphosphate carboxylase/oxygenase activase (Os11g0707000), and cysteine synthase (Os12g0625000) were isolated by RT-PCR from four-week-old *Oryza sativa* cv. Ilmi seedlings using the indicated primers (Table S1). The full-length cDNAs encoding these genes were cloned into the pET-DsRed expression vector which was previously used for other protein's expression and purification.

### *Agrobacterium*-mediated transformation of rice

The Agrobacterium strain LBA4404 harboring *MSRB2-Bar* or *MSRB2*-mini was introduced into embryogenic calli rice (*Oryza sativa* L. Japonica cv. Ilmi) by *Agrobacterium*-mediated transformation [31]. Callus induction, cocultivation with *A. tumefaciens*, and the selection of transformed calli were carried out as previously described by Sohn et al. [31]. Selected calli were subcultured 2 weeks on fresh 2N6 medium [31] with 250 mg/L cefotaxime and transferred to MSR medium [31] with 250 mg/L cefotaxime and 4 mg/L phosphinotricin for selection and regeneration for 4 weeks at 27°C under continuous light condition. The regenerated shoots were transferred to MSO medium [31] with 4 mg/L phosphinotricin for root induction for 4 weeks. The plantlets were then transplanted to a Wagner?pot (200 cm^2^) in a greenhouse for subsequent growth. Both the *MSRB2-Bar* and *MSRB2*-mini transgenic rice of T_3_ generation were used for further studies.

### Cellular localization of CaMSRB2

Ten µg of these constructs were introduced into the rice protoplasts (10^6^ cells/ml) using polyethylene glycol-mediated transformation [32]. The expression of the fusion constructs was monitored 24 h after transformation by a confocal laser scanning microscope (Carl Zeiss LSM 510). The filter sets used were excitation: 489 nm, emission: 508 nm and excitation: 558 nm, emission: 583 nm for GFP and DsRed, respectively.

### RNA isolation and RT-PCR

The total RNA was extracted from the leaves of the transgenic and WT rice plants using TRI REAGENT (Molecular Research Center, www.mrcgene.com) and cleaned up by the Qiagen RNeasy mini kit (Qiagen, www.qiagen.com). The cDNA templates were synthesized by RevertAid H minus M-MulV reverse transcriptase (Fermentas, www.fermentas.com). For the semiquantitative RT-PCR, the PCR amplifications were performed in 20 µl volumes with the following protocol: one cycle of 95°C for 2 min, 25 to 30 cycles of 94°C for 30 s, 60°C for 30 s, and 72°C for 30 s. The real-time quantitative RT-PCR analysis was carried out using 2× real-time PCR Pre-mix with EvaGreen (SolGent, www.solgent.com) according to the manufacturer's protocol. The thermal cycling and fluorescence detection were performed using a Stratagene Mx3000P Real-Time PCR machine and Mx3000P software v2.02 (Stratagene, (http://www.genomics.agilent.com). A melting curve analysis (55–65°C at a heating rate of 0.1°C s^−1^) was performed to ensure that only the required PCR product at a specific melting temperature was measured. The real-time PCR reactions were performed in triplicate for each cDNA sample. Following amplification, the experiment was converted to a comparative quantification (calibrator) experiment type and analyzed with the Mx3000P software v2.02 (Stratagene). The rice *tubulin* gene (Os11g0247300) was used as the endogenous control. All of the primer pairs are listed in Table S1.

### Methyl viologen treatment

The leaves were detached from four-week-old plants of the transgenic and WT lines and then submerged in liquid Murashige and Skoog (MS) medium (Murashige and Skoog 1962) containing 0.1 mM MV (Sigma, www.sigmaaldrich.com) in 0.1% Tween-20 [14] and exposed to continuous light (150 µmol protons m^−2^ S^−1^) at 28°C for 2 days. The sensitivity of the leaves to MV was judged visually. And then we quantified the chlorotic/necrotic areas per total area (%) using Image J software (Wayne Rasband, National Institute of Health, USA).

### Chlorophyll fluorescence under drought stress

The leaves were detached from four-week-old plants of the transgenic and WT lines and were air-dried at 25°C?under continuous light (100 µmol protons m^−2^ S^−1^) [33]. The *Fv/Fm* (arbitrary units) was measured by mini PAM (WALZ, www.walz.com) according to the time course. *Fv/Fm* indicates the potential maximal PSII quantum yield, and the value of healthy leaves is approximately 0.8 [34]. The results shown are the mean ± SE, n = 5. The experiments were conducted in three replicates.

### Drought treatment

The CaMSRB2-overexpression transgenic and WT rice (*O. sativa* L. Japonica cv. Ilmi) were examined using polyethylene glycol (PEG) as a preliminary drought test and then subjected to drought stress at their various stages.

To evaluate the drought tolerance of the seedlings, manually dehusked seeds were sterilized in 15% sodium hypochlorite (commercial bleach) for 10 minutes followed by three rinses in sterile distilled water in a laminar flow cabinet. The sterilized seeds were cultured in test tubes containing MS medium. The test tubes were placed in a growth chamber under fluorescent light and at an ambient temperature of 25±2°C. Four week old seedlings were cultured in the MS medium adjusted to 10% polyethylene glycol (PEG) (6000) for two days. PEG solutions were decanted and filled with MS medium and further cultured in the chamber for eight days. And then fresh weight and dry weight of the plants were measured.

For the drought treatment during vegetative stage, the sterilized seeds were directly planted at densities of nine seeds per 5-cm^3^ pot of the nursery bed soil. This tray was transferred into a plastic box filled with water and placed in a greenhouse under natural light (16-h-light/8-h-dark cycles) at an ambient temperature of 30±2°C. Four-week-old transgenic and WT plants were treated with drought stress by decanting all of resting water in the plastic box and ceasing irrigation for three days. The survival rate and chlorophyll index were measured after re-watering for two weeks. The survival rates were calculated from 18 plants for each genotype. The chlorophyll index from ten plants was measured using a chlorophyll meter (SPAD502).

For drought treatment at the reproductive stage, twenty sterilized seeds were grown in pots (200 cm^2^, 1 plant per pot) in a greenhouse under natural light (16-h-light/8-h-dark cycles) at an ambient temperature of 30±2°C. Drought stress was initiated at the panicle heading stage (15 days before heading). To induce drought stress, 12-week-old transgenic and WT plants were not watered for 0, 3, 5, 7, or 9 days. After the drought stress, the plants were irrigated to allow for recovery during the seed maturation stages. The resistance of the leaves to drought stress was judged visually. The soil moisture suction was recorded using a soil tensiometer (DIK-3023, Daiki Rika Kogyo Co., Ltd. Japan)

### Microarray Analysis

Four-week-old transgenic and WT plants were used for microarray analysis in triplicate. And these plants were treated with drought stress by withholding water for two days, which were used for microarray analysis in replicate. The expression profiling was conducted with the rice 3′-Tilling 135 k Microarray v3.0 manufactured by NimbleGen. The microarray was scanned with Genepix 4000 B (Axon,) preset with a 5 um resolution and for Cy3 signal. Signals were digitized and analyzed by Nimblescan (Nimblegen, U.S.A.). The data was normalized and processed with cubic spline normalization using quantiles to adjust signal variations between chips [35]. Probe-level summarization by Robust Multi-Chip Analysis (RMA) using a median polish algorithm implemented in NimbleScan was used producing calls files. The method identifies probes that are outliers in the overall behavior of the expression measured for a given gene and the contribution by those outliers is reduced in the reported gene expression level.

### Detection of MetSO-containing proteins in the leaf extracts

The leaves from WT and transformed rice that were treated with and without drought stress for 2 days were ground in liquid nitrogen. And then 6 g of powder was resuspended in 10 ml of lysis buffer (50 mM Tris pH 7.0, 100 mM NaCl, and protease inhibitor cocktail [Roche, www.roche.com]).The samples were then cleaned by successive centrifugations at 10,000×*g*. For the western blot analysis, 15 ug of the protein extracts obtained by using Bradford method (Bio-Rad, www.bio-rad,com) were separated on SDS-PAGE, and the proteins were transferred onto PVDF membranes by semidry electroblotting. The membrane was placed in 30 ml of blocking buffer (3% non-fat skim milk in PBS) for 1 hr and incubated with primary antibody, the Methionine Sulfoxide Polyclonal Antibody (Cayman), at a 1∶1,000 dilution in blocking buffer at 4°C overnight with gentle shaking. The blot was washed four times with PBS containing 0.2% tween-20 and incubated with an anti-rabbit secondary antibody (Goat Anti-Rabbt IgG-HRP, Santa Cruz, www.scbt.com) at a 1∶10,000 dilution in blocking buffer for 1 hour at room temperature with gentle shaking. After washing at least 3–4 times, the blots were developed using an ECL kit (Pierce).The band intensity was quantified using the Multi Gauge V2.3 program (Fujifilm).

### Production of recombinant proteins

These proteins were produced in the *E. coli* strain BL21 Star (DE3) (Invitrogen), incubated at 37°C until OD = 0.6, and induced by IPTG at 25°C for 5 hours. The cell was resuspended in 5 ml of lysis buffer [50 mM NaH2PO4, 300 mM NaCl, 10 mM imidazole (pH 8.0)], sonicated five times with 15 seconds burst and 45 seconds cooling on ice, and centrifuged at 14,000 g for 30 min at 4°C. The supernatant was incubated with 2 ml of Ni-NTA agarose (Qiagen) for 30 minutes, loaded into the empty column, and washed two times with 5 ml of lysis buffer puls 10 mM imidazole according to the manufacturer's instructions, The purified protein was eluted with 0.5 ml of lysis buffer puls 240 mM imidazole. 5 ug of the purified proteins was used for each experiment.

### In-gel digestion

The separated protein bands of interest were excised and washed twice with distilled water. The gels were then de-stained with 50 mM ammonium bicarbonate (ABC) in a 50% acetonitrile (ACN) aqueous solution and further sliced into sizes of approximately 1 mm^2^. The gels were subsequently dehydrated in 70% and 100% ACN, reduced with dithiothreitol (DTT), and alkylated with iodoacetamide (IAA). The gels were once again washed and dehydrated. After the gels were fully dried, they were rehydrated with 25 ng/µl trypsin in a reaction buffer (50 mM ABC, 0.1 mM CaCl_2_, pH 8.0, freshly prepared). Any remaining solutions were discarded from the gels, and 50 µl of additional reaction buffer was added. After incubation at 37°C overnight, 50 µl 10% formic acid in a 10% ACN solution was added, and the digested peptides were extracted by ultrasonication. The peptides were further extracted using 0.1% trifluoroacetic acid (TFA) in 50% ACN, 0.1% TFA in 70% ACN and finally 0.1% TFA in 100% ACN. The collected peptides were dried in a SpeedVac (Thermo Scientific, www.thermofisher.com) vacuum evaporator and reconstituted in 10 µl 0.1% formic acid in distilled water. The samples were centrifuged before injection.

### Nano-LC-MS/MS analysis

The prepared peptide samples were analyzed using a LTQ XL linear trap mass spectrometer (Thermo Scientific) equipped with a nano-HPLC system (Eksigent, www.eksigent.com). A 5-µl peptide sample was injected and loaded onto a C18 trap column (2.5 cm×100 µm i.d., Upchurch Scientific, Oak Harbor, WA, USA) using an auto-sampler. The trapped samples were eluted and separated through an in-line, homemade reverse-phase C18 micro column that was packed with a fused-silica nano-spray tip (column dimension, 10 cm×100 µm i.d.; particle size, 5 µm; pore size, 300 Å; Nanobaume). The peptides were eluted using the mobile phase gradients of solvents A and B (0.1% formic acid in HPLC water and 0.1% formic acid in ACN, respectively) with a flow rate of 200 nl/min. The gradient started with 2% solvent B and increased to 50% in 100 min; it then increased to 100% in 105 minutes. After 5 minutes of maintaining 100% solvent B (washing), the column was equilibrated to 98% solvent A and 2% solvent B for another 10 minutes. The eluted peptides were ionized by nano-spray with a voltage of 1.4 kV and subjected to mass spectrometry. The peptide ions were first analyzed with a full-MS scan in the range of 300-2000 *m/z*, and the 7 most intense ions from the full-MS scan were data-dependently selected for the CID tandem MS analysis (normalized collision energy of 35 for 30 ms). The dynamic exclusion parameters of the data-dependent scan were repeat count  = 2, repeat duration  = 30 sec, list size  = 300, exclusion duration  = 180 sec, low mass width  = 0.8, and high mass width  = 2.2.

### Statistical analyses

All statistical analyses were performed using Sigma plot v10 software.

## Results

### Characterization and localization of CaMSRB2

**Figure 1:**
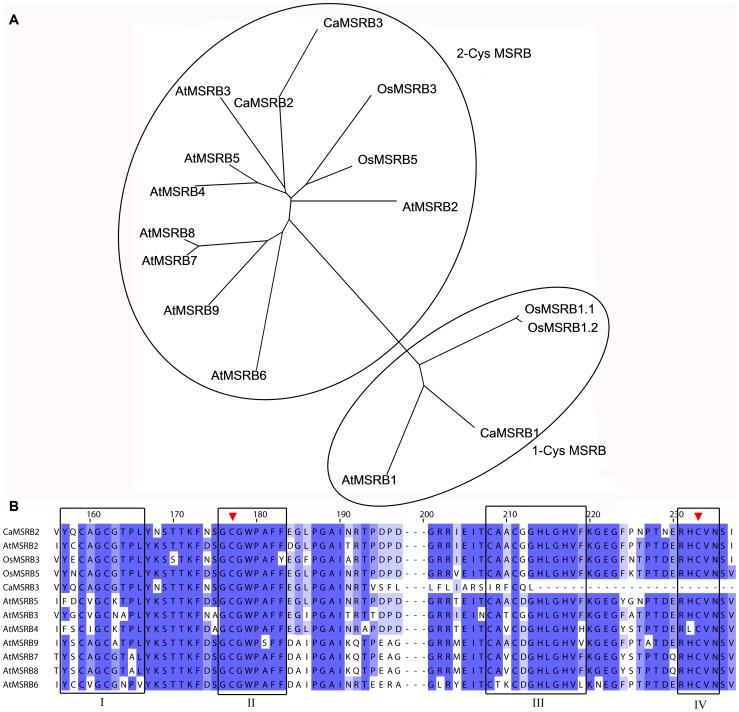
Phylogenetic and amino acid comparisons of the MSRBs. (A) Phylogenetic tree of the MSRBs from *Arabidopsis thaliana* (AtMSRB1: NP_564640, AtMSRB2: NP_567639, AtMSRB3: NP_567271, AtMSRB4: NP_192390, AtMSRB5: NP_192392, AtMSRB6: NP_192393, AtMSRB7: NP_567637, AtMSRB8: NP_193915, AtMSRB9: NP_567638), Oryza sativa (OsMSRB1.1: AK063588, OsMSRB1.2: AK111486, OsMSRB3: AK071730, OsMSRB5: AK068764), and *Capsicum annuum* (CaMSRB2: EF144171, CaMSRB1: EF144174, CaMSRB3: EF144173). The phylogenetic tree was constructed by ClustalW and Phylip package (Neighbor-joining method) with full-length sequences including transit peptides and visualized using TreeView. (B) The amino acid sequence alignment of the conserved SelR domain of the MSRBs from *Arabidopsis thaliana, Oryza sativa*, and *Capsicum annuum* was constructed using ClustalW (http://www.ebi.ac.uk/Tools/msa/clustalo/). Four putative MSRB signature motifs are boxed, and two conserved Cys residues are indicated with arrows.

Because overexpression of *CaMSRB2* in a dicot plant, tomato, leads to enhanced tolerance of oxidative stress [17], which results in increased resistance to pathogens, we predicted that the gene could also function in a monocot model plant, rice, because heterologous gene expression represents a potential approach to improve stress tolerance, avoiding endogenous mechanisms that often co-suppress the transgene of interest [36]. To examine the shared identity of the gene with other members from rice, Arabidopsis and pepper, 9, 4 and 3 genes were retrieved from RAP2, TAIR9 and CaEST DB, respectively, by BLAST analysis. From the phylogenetic tree that is shown in Figure 1A, the MSRBs were divided into 1-Cys type and 2-Cys type, which display one or two redox-active cysteines, respectively; the homology ranges from 63 to 98% within 1-Cys type MSRB sequences and 54 to 90% within 2-Cys type MSRB sequences, but only 28 to 45% between 1-Cys and 2-Cys type MSRBs sequences. CaMSRB2 contains the typical putative four conserved motifs in a SelR domain, which is a conserved 121 amino acid region that is the catalytic region of MSRBs [17]. Comparing the SelR domain alone, CaMSRB2 showed a high amino acid identity with AtMSRB2 (89%), OsMSRB3 (88%), and OsMSRB5 (87%) (Figure 1B). AtMSRB2 is localized to the chloroplast [13], and OsMSRB3 has a chloroplastic or mitochondrial targeting peptide (TargetP 1.1 Server, www.cbs.dtu.dk/services/TargetP/) and is predicted to be localized in the chloroplast or mitochondria. Interestingly, CaMSRB2 not only contains the putative basic residues (^42^KRRFR^46^) of a nuclear localization signal but also has the putative N-terminal chloroplast transit peptide sequence (^3^SQILKISPF11).

**Figure 2:**
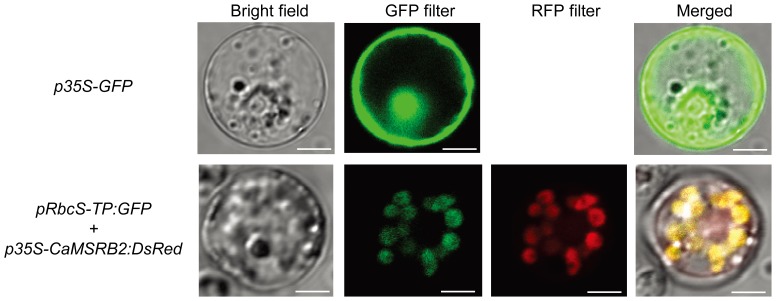
Subcellular Localization of CaMSRB2 by transient expression. The expression of CaMSRB2 fused in-frame to DsRed was driven by the *CaMV* 35S promoter in rice protoplasts and examined under a confocal microscope. GFP with an N-terminal chloroplast transit peptide was constructed under the control of the *RbcS* promoter as a chloroplast marker. White scale bar represents 5 um.

To test the exact cellular localization of OsMSRB3, OsMSRB5, and CaMSRB2 in rice, a DsRed fusion vector that was driven by the CaMV 35S promoter was constructed and transformed into rice protoplasts. As shown in Figure 2, CaMSRB2 fused N-terminally to DsRed was co-transformed with the known chloroplast marker, Rbc-TP::GFP [28]. The RFP image of the CaMSRB2-fused fluorescence signals was exactly aligned to the GFP-filtered Rbc-TP::GFP image, suggesting that CaMSRB2 is localized to the chloroplast. The GFP fluorescence spread throughout the cytoplasm and the nucleus when GFP alone was expressed as a negative control. In addition, the RFP-filter image of OsMSRB3 is clearly distinct from the nucleus locater, NLS::RFP, suggesting that the protein does not localize to the nucleus (Figure S1). The image is closer to the shapes of chloroplasts and also different from that of OsMSRB5, which is localized to the cytosol. Although we did not test the co-localization of OsMSRB3 with a chloroplast marker, the shapes of its florescent images are very similar to those of Rbc-TP::GFP and CaMSRB2::DsRED. The results suggest that CaMSRB2 proteins are targeted to the chloroplast and repair oxidized proteins accumulating in the chloroplast.

### Overexpression of the CaMSRB2 gene in rice

**Figure 3:**
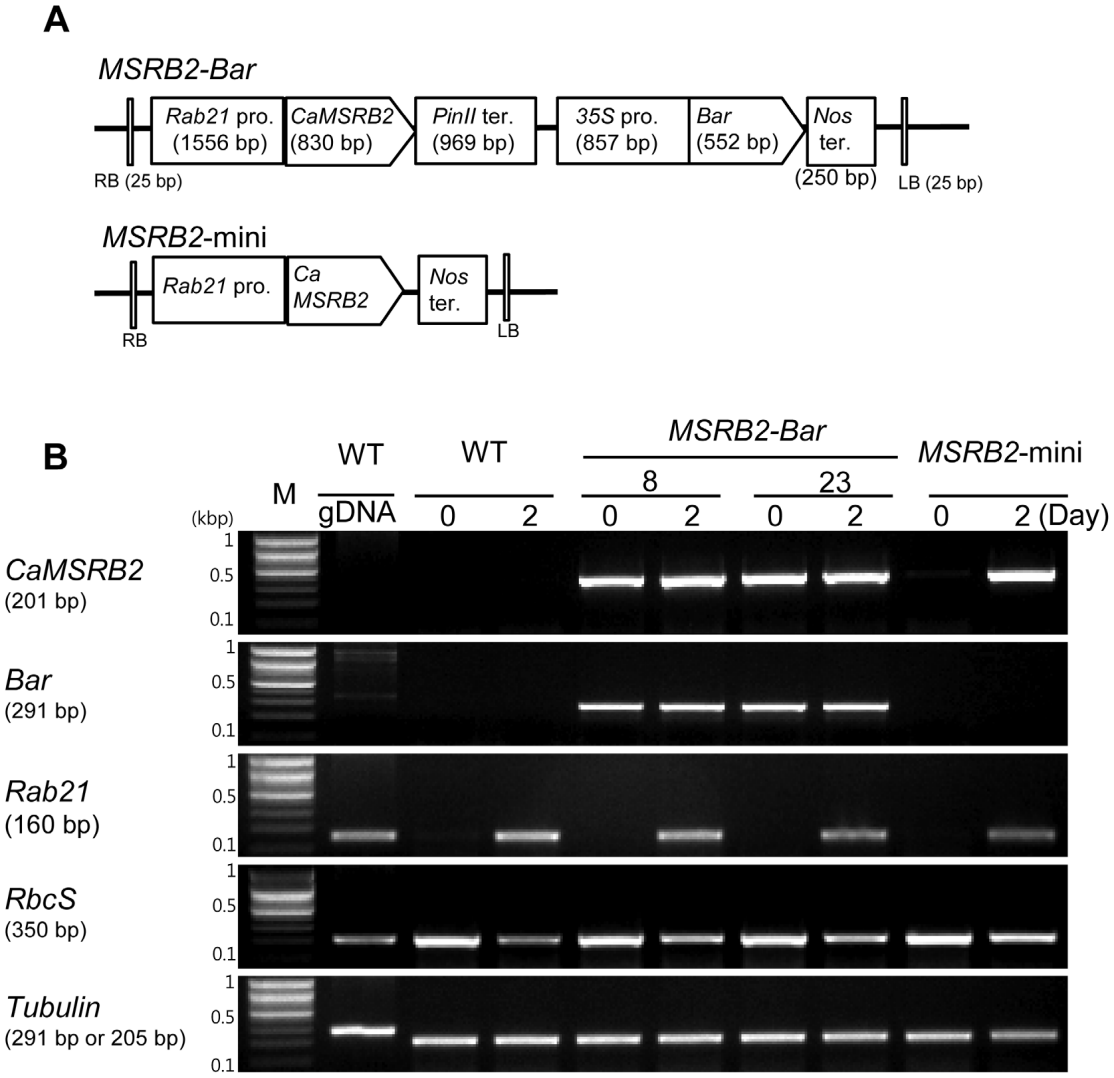
Production of *MSRB2-Bar* and *MSRB2*-mini transformed rice. (A) Schematic representation of the expression vectors that were used for rice transformation. *CaMSRB2*, pepper methionine sulfoxide reductase B2; *Rab* pro, rice responsive to ABA protein 21 promoter; *PinII*ter, potato proteinase inhibitor II terminator; *35S* pro, cauliflower mosaic virus 35S promoter; *Bar*, phosphinothricin acetyltransferase; *Nos*ter, nopaline synthesis terminator. (B) RT-PCR analysis of *CaMSRB2* gene expression in WT, *MSRB2-Bar* and *MSRB2*-mini plants under drought stress for 2 days. *Rab21* and *RbcS* were used as a positive and negative control for drought stress, respectively, and *tubulin* was used as an internal control.

To examine the role of CaMSRB2 in transgenic rice, we constructed two distinct plasmids for rice transformation. In the constructs (Figure 3A), *CaMSRB2* was expressed under the control of the promoter of rice *Rab21* (responsive to ABA protein 21), a gene known to be induced during the drought response [27], [37]. The *MSRB2-Bar* plasmid contains the bar gene as a selectable marker to identify the transgenic cells. The bar gene encodes phosphinothricin acetyltransferase, which converts phosphinothricin into the nontoxic acetylated form [38]. This gene was placed under the control of the *35S* promoter of the cauliflower mosaic virus. It has been reported that the presence of the marker gene causes the following negative side effects: (i) decreased ability of transgenic cells to proliferate and differentiate [39] and (ii) gene transfer by out-crossing within and between species [40]. Thus, we generated a minimal vector *MSRB2*-mini plasmid that contains the same *Rab21* promoter that was used in the *MSRB2-Bar* plasmid for the expression of CaMSRB2 but does not include the bar marker gene (Figure 3A).

Nineteen independent transgenic lines for *MSRB2-Bar* were obtained using the *Agrobacterium*-mediated transformation method [31] under bar selection conditions. Five independent transgenic lines from approximately 1000 plants for *MSRB2*-mini without bar selection were obtained using the *Agrobacterium*-mediated transformation method. The T-DNA flanking regions from 24 independent T_0_ transgenic lines (19 lines from *MSRB2-Bar*, and 5 lines from *MSRB2*-mini) were isolated by the adaptor-ligation PCR method [41], sequenced, and then analyzed by FSTVAL, a transgene flanking sequence validator [42] (Table S2). The lines 8 and 23, which harbor the transgenes as an intergenic single copy from the *MSRB2-Bar* vector, and a line harboring the *MSRB2*-mini vector were selected for further analysis. The RNA was extracted from four-week-old transgenic and wild type (WT) rice just before and 2 days after water withholding. The drought stress treatment was confirmed with the stress-indicative marker *Rab21* (Figure 3B). *CaMSRB2* in both *MSRB2-Bar* and *MSRB2*-mini transgenic rice was highly expressed after a drought stress of 2 days. Interestingly, *CaMSRB2* in *MSRB2-Bar* is induced even without drought stress treatment. We suspect that the plants are being affected by the constitutive *35S* promoter or stressed by the constitutive expression of bar under the *35S* promoter (Figure 3B, indicated by *Bar*). It has been shown some pathogenesis related (PR) genes such as PBZ1 and POS22.3 are slightly enhanced in 35S:bar-transgenic rice suggesting Bar gene or its gene product modulate some gene expression [43]. Indeed gene expression of some PR genes are fortified by glufosinate ammonium treatment followed by pathogen inoculation in the Bar rice. We tried to test the plant of *MSRB2-Bar* was influenced by the constitutive bar gene expression by measuring the growth or flowering time but we did not find any phenotypic differences in their appearances compared to those of wild type and *MSRB2*-mini.

### Response of *CaMSRB2-*transformed plants to oxidative and drought stress

**Figure 4:**
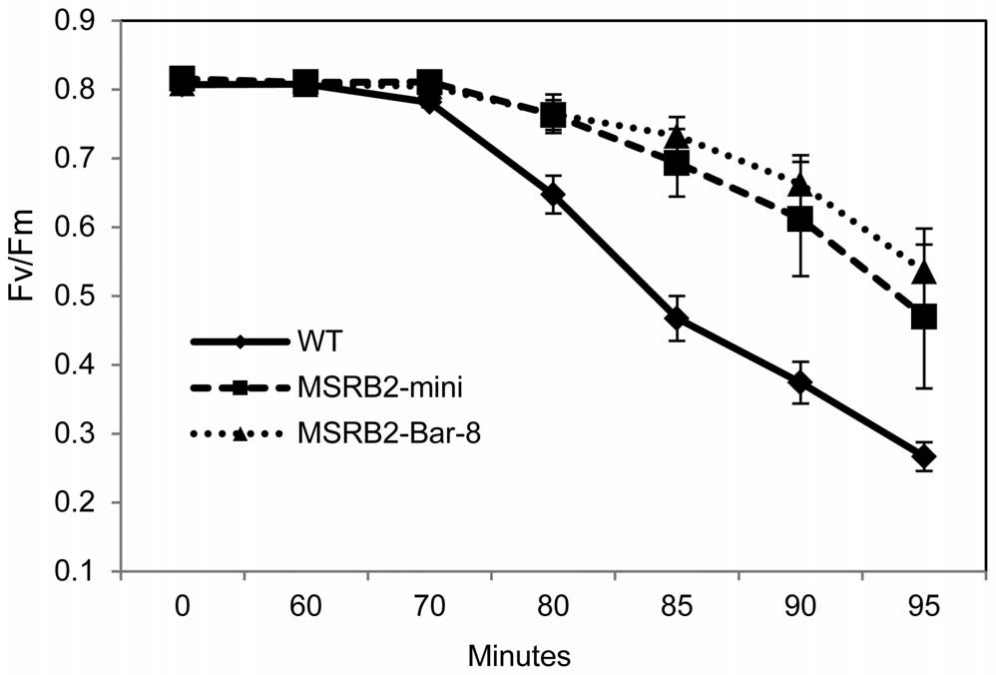
Changes in chlorophyll fluorescence during drought stress. The leaves were detached from four-week-old plants of the transgenic and WT lines and were air-dried at 25°C under continuous light (100 µmol protons m^−2^ S^−1^). The *Fv/Fm* value was measured by mini PAM according to the time course. The results shown are the mean ± SD, n = 5 replicates for each group. The experiments were representative of three independent experiments.

Both the *MSRB2-Bar* and *MSRB2*-mini transgenic rice in their T_3_ generation showed no phenotypic difference from WT under normal growth conditions (Figure S2). Because *CaMSRB2* has been shown to enhance the tolerance to oxidative stress in tomato plants [17], the *CaMSRB2-*transformed rice plants were tested for resistance to methyl viologen (MV), an oxidative stress-inducing agent. The MV ion reacts with the free electrons from photosystem I to give the free radical form. Chemically highly reactive superoxide is produced when the free radicals react with oxygen and attack the unsaturated membrane fatty acids, resulting in the disintegration of the cell membranes and tissues. When the detached leaves of the transgenic lines or of the WT were submerged in MV (0.1 mM), the chlorinated regions and block spots were significantly reduced compared to those of the control (Figure S3). Given the reports that the production of ROS is enhanced by the MV treatment, these data suggest that damage due to the ROS production is decreased in *MSRB2*-mini and *MSRB2-Bar* rice compared to that of WT. *MSRB2-Bar* rice seems to slightly better perform than those of *MSRB2*-mini in MV treatment. It remains to be answered that how bar gene expression influence in this reaction. It has been known that bar gene can deactivate the toxic effect of glufosinate ammonium by transferring Acetyl group to the chemical. Glufosinate ammonium deactivates irreversibly Gln synthetase, which results in an accumulation of toxic ammonium derived from photorespiration and inorganic nitrogen assimilation [44]. Accumulation of toxic ammonium disturbs electron transport systems of both chloroplasts and mitochondria and induces production of hazardous free radicals [45]. As described above bar might potentiate the readiness of drought response in the MSRB2-Bar-8 plant by modulating the genes involved in the reaction. We also investigated whether the expression of *CaMSRB2* was correlated with the drought response in the transgenic rice. To avoid the complications of interpretation due to genic and multi-copied transgenes, five plants (lines 4, 8, 14, 23, and 30) that harbor the transgene as a single copy in the intergenic regions were chosen for polyethylene glycol (PEG) as a preliminary drought tolerance indicator as described in methods (Figure S4). One line (line 29) with a single-copy transgene in the genic region was also included for comparison. Four-week-old seedlings were cultured in MS medium with 10% polyethylene glycol (PEG) (6000) for two days. The fresh weight (FW) of the plants that were treated with 10% PEG was measured, and their percentage weight was presented by comparison with the FW of the non-treated plants. Similarly, the percentage of dry weight (DW) was presented by comparing the weights of the plants with/without the PEG treatment. As shown Figure S4, the fresh weights of lines 4, 8, 14, 23, and 29 showed significantly increases. The dry weights of all of the lines except for line 29 showed an even higher significance (p-value 0.01). We examined the drought stress response of WT, *MSRB2*-mini, and *MSRB2-Bar-8* plants by measuring changes in the chlorophyll fluorescence with Mini PAM. The leaves from four-week-old plants were air-dried at 25°C under continuous light (100 µmol protons m^−2^ S^−1^). The *Fv/Fm* indicates the potential maximal PSII quantum yield, and the value of healthy leaves is approximately 0.8 [34]. The reduction in the values of *Fv/Fm* was considerably larger in WT than in either the *MSRB2*-mini or *MSRB2-Bar* plants throughout the time course (Figure 4), suggesting that the WT plants are likely to suffer from PSII photoinhibition more than are the transgenic plants. Both the *MSRB2*-mini and *MSRB2-Bar-8* plants were less damaged in the PSII system, although we cannot say that quantum yields increased by *CaMSRB2*.

**Figure 5:**
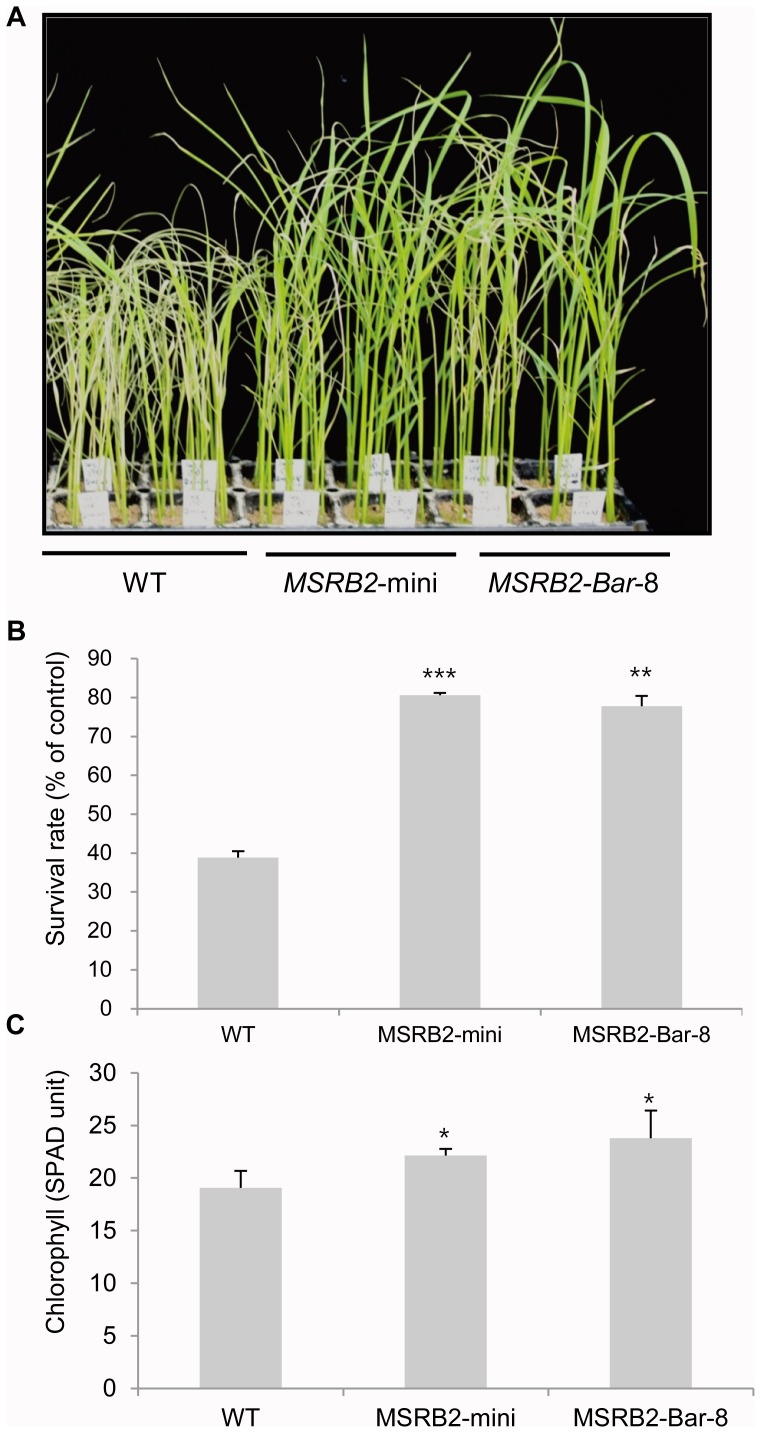
Response of CaMSRB2-transformed rice to drought stress. (A) Phenotypes of transgenic plants and WT under drought stress. Four-week-old plants were treated under drought stress conditions for 3 days. The pictures were taken after re-watering for 5 days. The experiments were representative of four independent experiments. (B) The drought tolerance was evaluated by the survival rate. The survival rates were calculated from 18 plants for each genotype. (C) The chlorophyll index from ten plants was measured using a chlorophyll meter (SPAD502). (B), (C) WT and transgenic plants were re-watered for two weeks. The results shown are the mean ± SD, n = 4 replicates for each group (***P<0.001, **P<0.01, and *P<0.05 compared to WT).

A drought tolerance test was then performed using whole plants in the soil as described in the methods. After a drought stress for 3 days, both the WT and transgenic lines were withered, and their leaves turned white. However, the growth of the transgenic lines was almost recovered after subsequent watering for 5 days (Figure 5A). In contrast, the growth of the WT plants was severely inhibited by the drought stress, and some plants never recovered. The data on the survival rate and chlorophyll index were measured after re-watering for two weeks. An average of 14 transgenic plants were recovered from 18 seedling plants after the drought stress (78% survival rate), but only 7 WT plants were recovered (39% survival rate) (Figure 5B). We also examined the chlorophyll index in ten plants using a chlorophyll meter. The chlorophyll index increased by 16 and 25% in the *MSRB2*-mini and *MSRB2-Bar-8* plants compared to WT, respectively (Figure 5C). These data indicate that the overexpression of *CaMSRB2* influenced both photosystems I and II and implicate an important functional role of *CaMSRB2* in conferring drought tolerance in rice. Additionally, the drought tolerance of the *MSRB2-Bar* (lines 8 and 23) plants was tested during their reproductive stage as well as their vegetative stage (Figure S2A–C). The water potential was monitored during the period of drought stress treatment, and re-watering was performed as is indicated in the methods (Figure S2D). The plants that were treated with stress for longer time periods showed the more severe symptoms. However, the *MSRB2-Bar* plants showed a better recovery than did the WT.

### Porphobilinogen deaminase is a putative target of MSRB2

**Figure 6:**
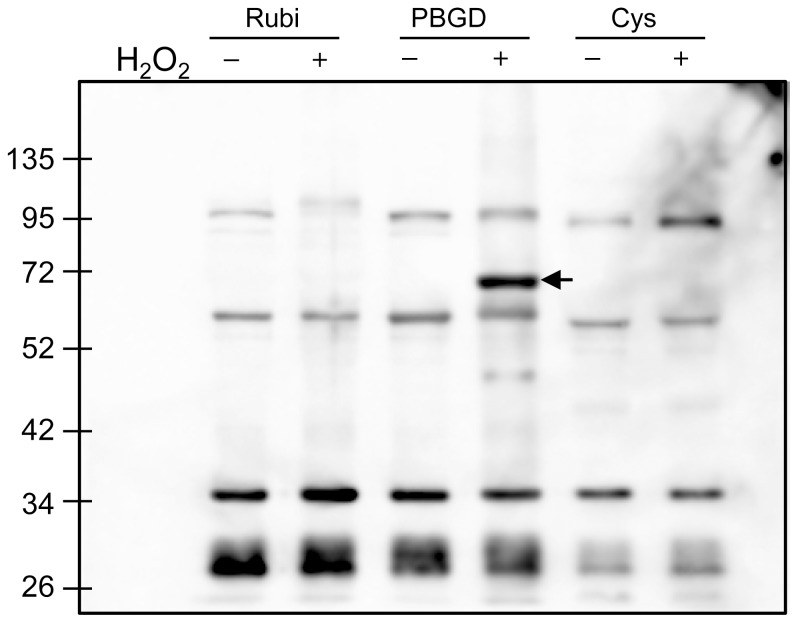
Detection of recombinant proteins containing methionine sulfoxide (MetSO) residues by western blotting. Rubi (Ribulose-bisphosphate carboxylase activase, Os11g0707000), PBGD (Porphobilinogen deaminase, Os02g0168800), and Cys (Cysteine synthase, Os12g0625000) were cloned into the pET-DsRed vector, expressed in *E. coli*, purified, and treated with 0.3% H_2_O_2_. These recombinant proteins containing MetSO were detected by western blotting with the methionine sulfoxide polyclonal antibody (Cayman). kDa, molecular mass indicators (in kDa The experiments were representative of three independent experiments.

As the transgenic lines overexpressing *CaMSRB2* were drought-tolerant, we reasoned that the MetSO of some proteins is more rapidly reversed by CaMSRB2. To test the possible targets of CaMSRB2 in the cell, we hypothesized that the MetSO of some proteins could be detected with an anti-MetSO antibody. The protein extracts were prepared from the WT plants and those lines overexpressing CaMSRB2 with/without drought stress. Equal amounts of protein extracts from each line were subjected to SDS-gel electrophoresis followed by western blot analysis using the anti-MetSO antibodies (Figure S5). The band intensities were quantified using the Multi Gauge V2.3 program (Fujifilm). A protein of approximately 40 kDa was significantly increased to approximately 2-fold in the WT plants under drought stress compared to the CaMSRB2-transformed rice (Figure S5). This result indicates that the WT plants are more sensitive to Met oxidation under drought stress than are the transgenic plants. To identify the oxidized proteins, the protein bands of approximately 40 kDa were excised from the SDS-PAGE gel and subjected to Nano-LC-MS/MS spectrometry analysis (Table S3). We identified 33 proteins with molecular weights ranging from 35 to 40 kDa, among which we chose three chloroplast genes to characterize more extensively: porphobilinogen deaminase (PBGD, Os02g0168800), ferredoxin-NADP reductase (Os06g0107700), and dihydrodipicolinate reductase 1 (Os02g0436400). We also chose ribulose bisphosphate carboxylase/oxygenase activase (Os11g0707000) and cysteine synthase (Os12g0625000) with molecular weights of 51 and 34 kDa and which are localized to the chloroplast and cytosol, respectively. The ORFs of these genes were amplified, fused to *DsRed::His*, and expressed in *E. coli*. Three proteins, ribulose-bisphosphate carboxylase activase, porphobilinogen deaminase, and cysteine synthase, could be expressed and solubilized under our experimental conditions. However, other proteins could not be expressed or solubilized. These solubilized proteins were purified using a Ni-NTA column and subjected to oxidation with 0.3% H_2_O_2_. These proteins were resolved on a 10% polyacrylamide gel and transferred to a nylon membrane. The additional bands were considered endogenous proteins in *E. coli* and did not change regardless of H_2_O_2_. Compared to these bands, the band intensity of the DsRed-fused PBGD (∼70 kDa) was strongly enhanced by H_2_O_2_ oxidation when detected by the anti-MetSO antibody on the membrane, but ribulose-bisphosphate carboxylase activase and cysteine synthase did not show any enhancement (Figure 6).

**Figure 7:**
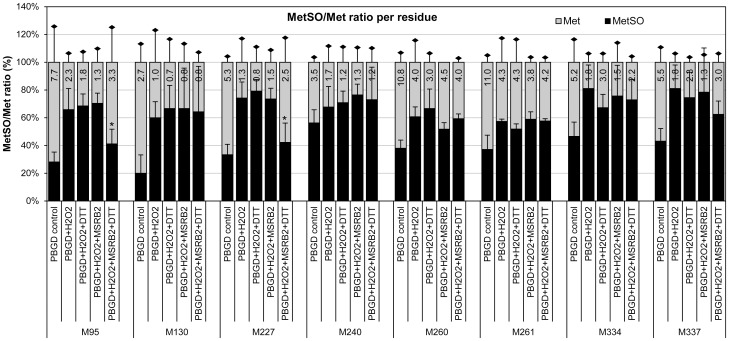
MetSO/Met ratio in individual methionine residues of PBGD. The gray and black bars represent the Met and MetSO residues, respectively. The MetSO content is expressed as the percent of MetSO in relation to the each Met residues (Met + MetSO). The figures that shown are in the bar graph represent the absolute numbers of Met or MetSO. The results shown are the mean ± SD, n = 3 replicates for each group (*P<0.05 compared to PBGD + H_2_O_2_ + MSRB2 value).

We further investigated which Met of PBGD might be the target of the CaMSRB2 enzymes. The purified recombinant PBGD was treated with 0.3% H_2_O_2_, further incubated with CaMSRB2 in the presence of dithiothreitol (DTT), a reducing agent, and subjected to nano-LC-MS/MS analysis after trypsin digestion (Figure S6). The catalytic activity of the MSRBs has been studied using protein-bound MetSO substrates and DTT or thioredoxin (TRX) as electron donors. The GmMSRB2 and GmMSRB4 proteins showed MSRB activity toward protein-bound MetSO with either DTT or TRX as reductants [46]. The percent Met oxidation increased (20–50%) in all of the Met residues with the addition of H_2_O_2_ (Figure 7). However, the addition of CaMSRB2 and DTT together caused a significant decrease in the oxidized Met-95 and Met-227 residues, while the other Met residues decreased marginally. These data suggest that the methionine residues of PBGD might be oxidized by stress and could then be reduced by CaMSRB2 in the presence of a reducing agent.

### Microarray Analysis of *CaMSRB2-*transformed plants

As PBGD is involved in tetrapyrrole biosynthesis, and evidence suggests that the accumulation of the ProtoIX and a Mg-porphyrin intermediate may be involved in the complex network controlling the stress-responsive genes [47], we hypothesized that *CaMSRB2* expression could affect gene expression during drought stress. To identify genes with altered expression levels in MSRB2, a microarray analysis was performed to compare the expression profiles between the transgenic (*MSRB2*-mini and *MSRB2-Bar*) and WT plants under normal and drought-stress conditions (GenBank accession number GSE41204). The profiling was conducted on a 135 K Rice Genome Microarray (GreenGene Biotech, www.ggbio.com), covering 31439 rice genes.

Under normal conditions, the minimal number of changes of down- or up-regulated genes was found and compared to those plants that were treated with drought stress (see below). A total of 20 and 54 genes were down- and up-regulated, respectively, in the transgenic compared to WT plants (P value <0.05, Figure S7). Among these genes, we found 14 and 22 genes to be down- or up-regulated, respectively, more than 2-fold in calli compared to leaves (Table S4). Tissue culture has been heavily used to generate transgenic crops such as rice and maize. In regenerated rice, many single-nucleotide polymorphisms (SNPs) and insertions and deletions (indels) were detected in addition to the transposition of Tos17 [48], [49]. This somaclonal variation can cause differential gene expression in transgenic plants.

**Figure 8:**
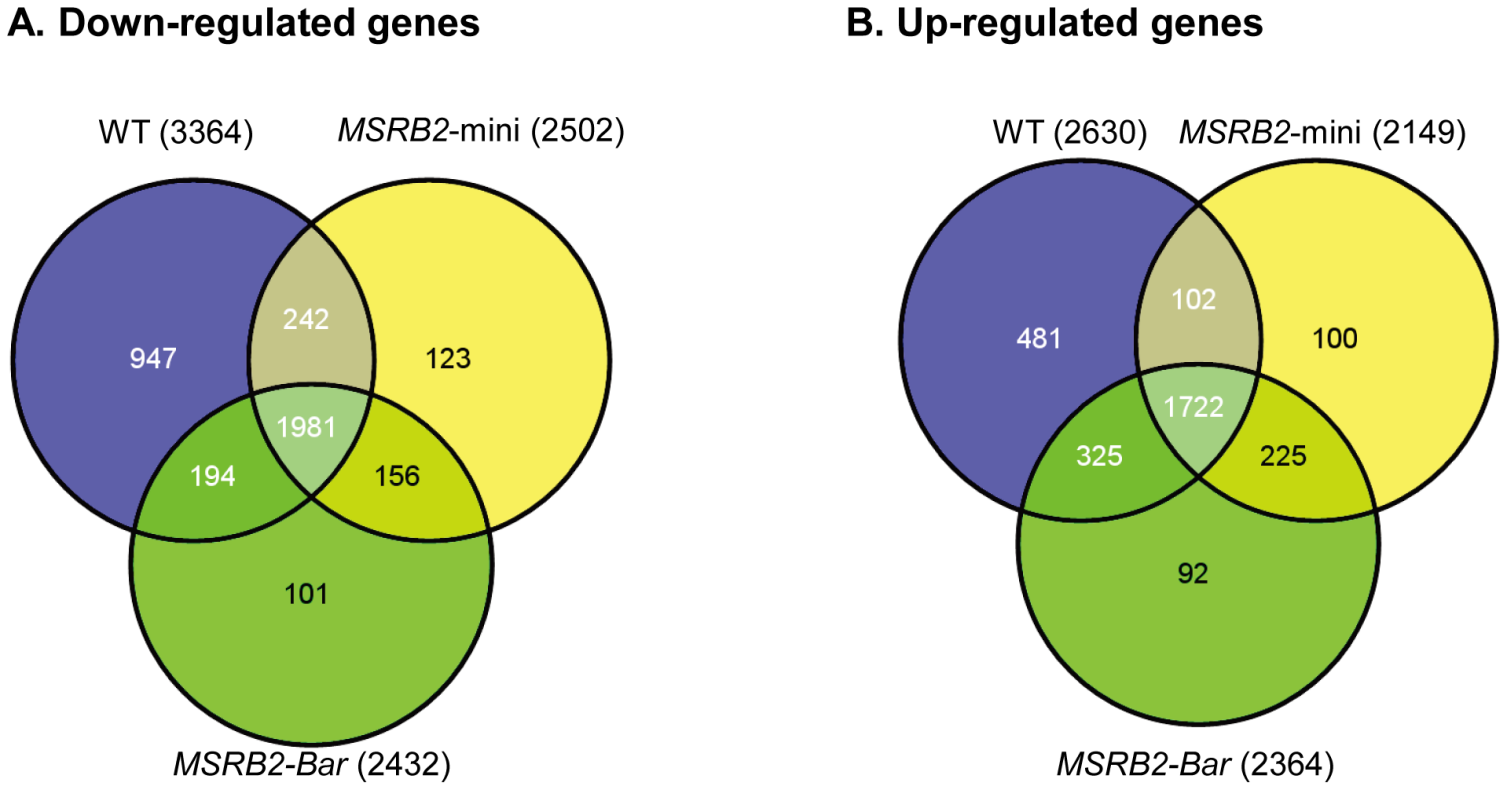
Venn diagrams of differentially expressed genes. Blue, yellow, and green represent the genes that were down- (A) or up-regulated (B) more than 2-fold in WT and transgenic plants after drought treatment compared to WT plants under normal conditions, respectively. A total of 1,981 and 1,722 genes were commonly down- or up-regulated in both WT and transgenic plants under drought stress, respectively. A total of 947 and 481 genes were only down- or up-regulated in the WT plants under drought stress, respectively.

When the plants were treated with drought stress, 3364, 2502, and 2432 genes were down-regulated more than 2-fold (P value <0.05) in the WT, *MSRB2*-mini, and *MSRB2-Bar* plants, respectively, compared to WT plants under normal conditions. The number of genes, 2630, 2149, and 2364, were up-regulated more than 2-fold (P value <0.05) in the WT, *MSRB2*-mini, and *MSRB2-Bar* plants, respectively, compared to WT plants under normal conditions. Although the WT plants shared 1981 down- and 1722 up-regulated genes in common with the transgenic plants under drought stress, the WT plants also had 942 differentially down-and 481 differentially up-regulated genes that were not detected at significant levels in the transgenic plants (Figure 8). The physiological role of the products of those genes that were significantly induced or repressed in the WT and transgenic plants under drought stress compared to control conditions was visualized with the software MapMan (Figure S8) [50]. Under drought stress, we found a highly significant down-regulation of genes that code for proteins that are involved in the photosynthetic light reactions, especially those of photosystem II. Gene repression was furthermore found for isoprenoid metabolism and a number of lipid and amino acid metabolism bins, especially the synthesis of ribosomal proteins (Table 1). These results are consistent with a previous study that found drought stress to lead to the down-regulation of some photosynthetic genes and metabolic components [51], [52].

**Table 1:** Statistical analysis of up- and down-regulated genes in MapMan BINs.

			common in 2d/WT0d			only in WT2d/WT0d			only in mini2d/WT0d			only in Bar2d/WT0d		
bin	name	n	p-value	down	up	p-value	Down	up	p-value	down	up	p-value	down	up
1	PS	54	**0.0019**	16	4	**0.00004**	30	2	0.2196	0	1	0.3062	1	0
1.1	PS.lightreaction	37	**0.0067**	12	4	**0.0009**	20	0	—	0	0	0.3062	1	0
1.1.1	PS.lightreaction.photosystem II	17	**0.0125**	6	2	**0.0006**	9	0	—	0	0	—	0	0
1.1.1.2	PS.lightreaction.photosystem II.PSII polypeptide subunits	11	**0.0223**	5	2	0.0831	4	0	—	0	0	—	0	0
1.1.6	PS.lightreaction.NADH DH	4	**0.0414**	3	0	0.6756	1	0	—	0	0	—	0	0
3.2	minor CHO metabolism.trehalose	7	**0.0391**	1	4	0.6104	1	0	—	0	0	0.9785	1	0
8	TCA/org. transformation	14	**0.0493**	1	4	0.7016	4	3	0.2544	2	0	—	0	0
10	cell wall	81	**0.0269**	51	20	**0.0369**	2	5	0.519	1	0	0.3240	2	0
11	lipid metabolism	106	**0.0349**	44	26	0.6425	21	12	0.0615	3	0	—	0	0
11.1.10	lipid metabolism.FA synthesis and FA elongation.beta ketoacyl CoA synthase	8	**0.0125**	6	1	0.1085	1	0	—	0	0	—	0	0
11.8.10	lipid metabolism.'exotics' (steroids, squalene etc).phosphatidylcholinesterol O-acyltransferase	7	**0.0111**	4	0	0.1826	1	2	—	0	0	—	0	0
13.1.3	amino acid metabolism.synthesis.aspartate family	12	**0.0461**	3	0	0.0745	8	0	0.0964	1	0	—	0	0
13.2.3	amino acid metabolism.degradation.aspartate family	4	**0.0496**	3	0	0.1482	0	1	—	0	0	—	0	0
16	secondary metabolism	88	**0.0229**	43	21	0.9139	12	6	0.6473	2	2	0.3209	2	0
19	tetrapyrrole synthesis	18	**0.0331**	12	2	0.1117	4	0	—	0	0	—	0	0
20.1	stress.biotic	107	0.8984	40	36	0.6964	15	10	0.1636	0	2	**0.0456**	0	4
20.2	stress.abiotic	87	0.1387	27	41	**0.0114**	5	8	0.3663	1	3	0.2416	2	0
20.2.1	stress.abiotic.heat	40	**0.0053**	7	24	0.1684	3	4	0.8343	1	1	—	0	0
20.2.2	stress.abiotic.cold	5	**0.0256**	0	4	—	0	0	0.3885	0	1	—	0	0
23.4	nucleotide metabolism.phosphotransfer and pyrophosphatases	6	**0.0296**	0	2	0.6559	2	2	—	0	0	—	0	0
26.1	misc.cytochrome P450	66	**0.0090**	33	19	0.6202	7	3	0.9072	1	0	0.7825	2	1
26.12	misc.peroxidases	23	**0.0000**	19	2	0.1396	0	1	—	0	0	0.1840	1	0
26.19	misc.plastocyanin-like	12	**0.0363**	3	8	—	0	0	—	0	0	0.3742	1	0
26.28	misc.GDSL-motif lipase	18	**0.0304**	10	1	0.4586	3	3	0.1095	1	0	—	0	0
27.1	RNA.processing	43	**0.7397**	15	8	0.0143	14	1	0.8498	3	2	—	0	0

			common in 2d/WT0d	only in WT2d/WT0d	only in mini2d/WT0d	only in Bar2d/WT0d								
bin	name	n	p-value	down	up	p-value	Down	up	p-value	down	up	p-value	down	up
27.3.3	RNA.regulation of transcription.AP2/EREBP, APETALA2/Ethylene-responsive element binding protein family	19	**0.0045**	1	11	**0.0031**	0	5	—	0	0	0.7649	1	1
27.3.23	RNA.regulation of transcription.HSF,Heat-shock transcription factor family	9	**0.0208**	0	7	—	0	0	0.1358	0	1	0.5298	0	1
27.3.25	RNA.regulation of transcription.MYB domain transcription factor family	44	**0.0102**	24	8	0.5480	11	1	—	0	0	—	0	0
28	DNA	85	**0.0113**	37	15	0.5452	19	6	0.6418	4	2	0.5842	1	1
28.1	DNA.synthesis/chromatin structure	30	**0.0132**	0	7	0.9780	12	5	0.9274	3	2	0.3742	1	0
29.2	protein.synthesis	54	**0.0162**	24	8	**0.0080**	16	1	0.2804	2	2	0.5298	0	1
29.3.2	protein.targeting.mitochondria	7	**0.0251**	0	5	0.2041	0	1	0.1789	1	0	—	0	0
29.6	protein.folding	22	**0.0188**	12	3	0.7872	4	1	0.2082	1	0	0.6407	0	1
29.5.1	protein.degradation.subtilases	11	**0.0368**	6	2	0.4264	1	1	—	0	0	0.2122	0	1
29.1	protein.aa activation	30	0.0541	18	2	0.6578	5	1	**0.0378**	3	0	0.9000	1	0
29.2.1	protein.synthesis.ribosomal protein	34	**0.0093**	16	2	0.0607	12	1	0.1957	1	1	0.5298	0	1
29.2.1.1	protein.synthesis.ribosomal protein.prokaryotic	19	**0.0015**	10	0	0.1873	8	0	—	0	0	0.5298	0	1
30.3	signalling.calcium	44	**0.0018**	12	25	0.0294	0	5	0.6356	0	1	0.8294	1	0
31.1	cell.organisation	78	**0.0340**	38	12	0.6365	17	9	0.2733	1	0	0.2155	1	0

A bin containing a significantly higher number of up- or down-regulated genes are prited in bold.

**Figure 9:**
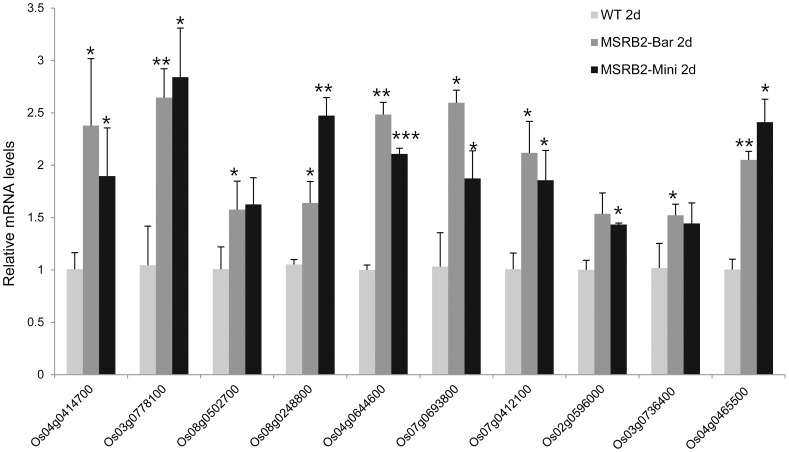
Expression of nine genes in the WT and CaMSRB2-transformed rice as detected by real-time PCR. The y-axis shows the relative expression level normalized to that of the WT plants that were grown under drought conditions for 2 days. Os04g0414700 (photosystem I PsaO domain-containing protein), Os03g0778100 (photosystem-1 F subunit), Os08g0502700 (pyridoxal phosphate-dependent transferase), Os08g0248800 (aspartate carbamoyltransferase 3), Os04g0644600 (esterases and lipases epoxide hydrolase family protein), Os07g0693800 (fatty acid desaturase), Os07g0412100 (granule-bound starch synthase Ib, chloroplast precursor), Os02g0596000 (rhodanese-like domain-containing protein), Os03g0736400 (methylase putative domain-containing protein), and Os04g0465500 (GCN5-related N-acetyltransferase domain-containing protein). The results shown are the mean ± SD, n = 3 replicates for each group (^***^ *P*<0.001; **P<0.01; and *P<0.05 compared to WT). The experiments were representative of two independent experiments.

Drought stress strongly down-regulated the photosynthesis genes in both the transgenic and the WT plants. However, the number of down-regulated genes was, surprisingly, higher in the WT plants than in the transgenic plants (Table 1). Among these chloroplast-targeting genes, 10 genes were randomly selected for further analysis and validation of microarray results using quantitative RT-PCR: Os04g0414700 (Photosystem I PsaO domain-containing protein), Os03g0778100 (Photosystem 1 F subunit), Os08g0502700 (Pyridoxal phosphate-dependent transferase), Os08g0248800 (Aspartate carbamoyltransferase 3), Os04g0644600 (esterases and lipases epoxide hydrolase family protein), Os07g0693800 (Fatty acid desaturase), Os07g0412100 (Granule-bound starch synthase Ib, chloroplast precursor), Os02g0596000 (Rhodanese-like domain-containing protein), Os03g0736400 (methylase putative domain-containing protein), and Os04g0465500 (GCN5-related N-acetyltransferase domain-containing protein). The real-time quantitative RT-PCR results were consistent with those of the microarray analysis. The chosen genes showed elevated expression levels in the transgenic lines under drought stress compared with those of the WT plants (Figure 9). The results suggest that a Pepper *MSRB2* gene confers drought resistance to rice through the protection of pathways in the chloroplast. PBGD might be a key regulator controlling these gene expression patterns.

## Discussion

### Overexpression of CaMSRB2 confers tolerance to drought by maintaining chloroplast function in transgenic rice

A phylogenetic analysis including genes from rice, Arabidopsis and pepper showed that 2-Cys type MSRBs are closely related to each other, ranging from 54 to 90%. Although they belong to one group, previous reports have shown that their localizations are varied: AtMSRB2 is localized to the chloroplast, and AtMSRB4 and AtMSRB5 are predicted to be cytosolic [13]. Indeed, OsMSRB5 is localized to the cytosol and OsMSRB3 is predicted to be localized to the chloroplast or mitochondria. This result is interesting in that CaMSRB2 has been previously shown to be localized to the nucleus and cytosol in tobacco protoplasts [17], but it localizes to the chloroplast in the rice protoplast. This result raises the possibility that the proteins in the same group have evolved through specified mechanisms, depending on whether the plant is a monocot or a dicot. Additionally, most pathogenicities are related to gene function in the cell membrane, cytosol, and nucleus, suggesting that the localization of the protein is also an important factor for gene function. Similarly, rice hexokinase 10 (OsHXK10), a key player in sugar signaling and metabolism, has been shown to be differentially localized in the cytoplasm and mitochondria in a tobacco expression system [53], [54]. Other members of HXK, OsHXK5 and OsHXK6 have recently been reported to retain a dual targeting ability for the mitochondria and the nucleus and to function as part of a glucose sensor in the cell [55]. The involvement of many transcription factors in signal transduction that are dual localized in both the nucleus and the cytosol has been well-documented, and a motif of the nuclear localization signal (NLS) may or may not be critical [56]. These data suggest that the localization of proteins could be flexible in the cell and that motifs such as the NLS and signal peptides are differentially interpreted depending on the cellular signals for their targeting.

The differential cellular targeting of the same gene predicts that the *CaMSRB2* functions could vary between tomato [17] and rice. In the initial experiment, we tested whether the overexpression lines of CaMSRB2 are resistant to *Magnaporthe grisea*, a rice blast fungus (data not shown). However, the degree of symptoms of transgenic rice did not show any difference from those of the controls. Interestingly, the transgenic lines showed a better performance under abiotic stresses. The MV treatment that produces ROS in PSI and the Fv/Fm analysis that is used to investigate the light harvesting and electron transport from PSII strongly suggest that *CaMSRB2* confers drought resistance in rice (Figure 4, Figure S3). This result is further confirmed by the survival rate and chlorophyll index after the re-watering of the WT and transgenic lines for 2 weeks (Figure 5B, 5C). Most of the transgenic lines showed recovery, while the WT did not (Figure 5A).

### PBGD is a substrate of *CaMSRB2* in rice

A putative target of *CaMSRB2* was further sought by immunoblotting the plant extracts that were treated with drought stress using an anti-MetSO antibody. A GC-Mass analysis and *in silico* search suggested that several proteins could be the target proteins. Tests of *CaMSRB2* reduction using those cloned genes suggest that *PBGD* is a putative target of *CaMSRB2*. Thus, the *PBGD* that was expressed in *E. coli* was oxidized in the presence of H_2_O_2_ and was reduced by *CaMSRB2* with DTT. The trypsin digestion of the protein followed by GC-Mass suggested that the Met-95 and -227 of PBGD could be imminent targets of *CaMSRB2*.

Methionine oxidation is one of the most common protein posttranslational modifications. In the western blot with the anti-MetSO antibody, we expected multi-bands in the transgenic plants compared to the WT under drought stress. However, we detected one band with a size of 34–42 kDa that was increased in the WT compared to the transgenic plants (Figure S5). The Met in the total amino acid composition of rice is only ∼3% [57], while the percentage of the surface-exposed Met is even lower. Therefore, most of the Met residues are predicted to be buried within the molecular structure of a protein, thereby making them more difficult to detect by western blotting using the anti-MetSO antibody [58]. There may be several other substrate proteins of CaMSRB2 in the chloroplast, but keeping PBGD, a chloroplast-localized protein, in good shape and active may be especially important because PBGD is so crucial for photosynthetic cell differentiation and for its roles in light and ROS homeostasis [59]. A *pbgd* mutant displayed a defect in the photosynthetic pigment accumulation and a reduction in ROS scavenging and is, therefore, more likely to be sensitive to ROS-induced damage [59]. These results from immunoblotting using a methionine sulfoxide antibody and nano-LC-MS/MS spectrometry show that PBGD is a substrate of *CaMSRB2* (Figure 6).

Initially, 5 proteins (ribulose-bisphosphate carboxylase activase [Os11g0707000], porphobilinogen deaminase [PBGD, Os02g0168800], ferredoxin—NADP reductase [Os06g0107700], dihydrodipicolinate reductase 1 [Os02g0436400], and cysteine synthase [Os12g0625000]) were selected and expressed in *E. coli*. Many enzymes are involved in the photosystem and CO2 fixation, especially Ferredoxin-NADP reductase, the last enzyme in the transfer of electrons during photosynthesis from photosystem I to NADPH. As the electrons are transferred from ferredoxin to NADP, an unbalanced electron transfer could result in ROS production, and the enzyme could be the primary target of MSRB. However, Ferredoxin-NADP reductase could not be confirmed because it was expressed in a non-solubilized form. As most of these enzymes are directly or indirectly involved in the photosystem, the targets of MSRB2 remain to be determined.

### Reduction of PBGD by *CaMSRB2* may be one important function involved in drought tolerance

To coordinate the genes encoding organellar proteins in different cellular compartments of the plant cell, such as the chloroplast and mitochondria, plants have evolved retrograde (organelle-to-nucleus) signaling [60], [61]. Biochemical and genetic research have demonstrated the changes in the redox status or energy balance of the chloroplast to the nucleus through redox-controlled protein phosphorylation and sensory systems [19]–[21]. ROS, such as superoxide, H_2_O_2_, and ^1^O_2_, also accumulate when the plant is exposed to excess light. Signaling pathways of these ROS seem to be distinctive, yet the cell is similarly damaged [22], [23]. Porphyrin may also be viewed as an attractive candidate for chloroplast molecules in the organelle-to-nucleus signaling pathways [62]. In a previous report, tetrapyrroles were found to be involved in the complex network controlling the stress-responsive genes and were greatly reduced in plants following drought treatment [47]. The transgenic plants overexpressing *Myxococcus xanthus* protoporphyrinogen oxidase showed significantly improved drought tolerance and increased nucleus-encoded photosynthetic genes, such as Lhcb1, Lhcb6, and Rubisco [47]. *PBGD* catalyzes the deamination and polymerization of four porphobilinogen (PBG) molecules into hydroxymethylbilane and functions early in the tetrapyrrole biosynthesis pathway leading to chlorophyll and heme [63]. CaMSRB2 reverts the oxidized form of *PBGD* to a reduced form to protect chlorophyll synthesis under drought stress. In an effort to identify genes that are involved in retrograde signaling using gun mutants, Mg-ProtoIX was shown to function as the trigger for repressing nuclear gene expression, and the molecules accumulated less in gun2 and gun5, resulting in the gun phenotype [24], [64]. Knock-out mutants of PBGD and the D-subunit of Mg-chelatase in the tetrapyrrole pathway also displayed the gun phenotype. Under normal conditions, deactivated enzymes that are involved in tetrapyrrole or chlorophyll synthesis might be recovered through the actions of enzymes such as MSR. However, prolonged abiotic stress could produce a loss of control of the monitoring systems and could produce excess Mg-ProtoIX, resulting in retrograde signaling.

The genes of the tetrapyrrole synthesis BIN (Table 1, Figure S9A) were significantly down-regulated in both the transgenic and WT plants under drought stress. Moreover, four genes in the tetrapyrrole synthesis pathway involving PBGD were down-regulated only in the WT plants under drought stress, but not in the transgenic plants (Figure S9B). Our results suggest that *CaMSRB2* might stabilize PBGD under drought conditions and contribute to maintaining tetrapyrrole metabolites.

At a glance, it is seemingly contradictory that PBGD is a target of MSRB under abiotic stress when strong gene down-regulation is needed along with ROS or metabolite byproducts to shut down gene expression. In this regard, endogenous MSRBs might function in the early phase of drought stress. If the plant is exposed to prolonged or unendurable stress, the chlorophyll synthesis itself could be harmful and the down-regulation of MSRBs would be required. Indeed, in rice, both OsMSRB3 and OsMSRB5 are down-regulated by drought stress (Figure S10).

## Conclusions

In conclusion, rice transgenic plants overexpressing the pepper CaMSRB2 gene perform better against drought stress compared to their WT counterparts. Several tests indicate that transgenic lines show less oxidative stress symptoms and a strengthened PSII quantum yield under stress conditions, and increased survival rate and chlorophyll index after the re-watering. In an effort to identify the target proteins, PBGD was identified as a putative target of CaMSRB2 in an in vitro test. Expression profiling analyses of the overexpression lines also suggest that the photosystems are less severely affected by drought stress, raising a possible function of MSRB in affecting retrograde signaling through PBGD. Our results indicate that CaMSRB2 might play an important functional role in chloroplasts for conferring drought stress tolerance in rice.

## Supporting Information

Figure S1 **Subcellular Localization of** ***CaMSRB3*** **and** ***CaMSRB5*** **by transient expression.** The expression of *CaMSRB3* and *CaMSRB5* are fused in-frame to the DsRed and their expression was driven by the CaMV 35S promoter in protoplasts of rice. The signals were examined under a confocal microscope. As a control for nucleus *NLS::RFP* were expressed in protoplasts of rice. These results showed *OsMSRB3*, *OsMSRB5* are localized in chloroplast and cytosol, respectively.
(TIF)

Figure S2 **Growth of CaMSRB2-overexpressing rice plants upon drought treatment.** (A) CaMSRB2-overexpressing lines (lines 8 and 23) and WT plants grown under normal conditions. (B) Twelve-week-old transgenic and WT plants that were not watered for 5 days. (C) Twelve-week-old transgenic and WT plants that were not watered for 7 days. (D) Measurement of soil water loss under drought stress. The soil moisture suction was recorded using a soil tensiometer (DIK-3023). The experiment was representative of three independent experiments.
(TIF)

Figure S3 **Response of CaMSRB2-transformed rice to an oxidative stress.** The detached leaves of wild-type, *MSRB2-Bar* and *MSRB2*-mini plants were incubated in MS medium containing 0.1 mM methyl viologen (MV) for 50 h. The resistance of the leaves to oxidative stress was judged visually. And then we quantified the chlorotic/necrotic areas per total area (%) using Image J software (Wayne Rasband, National Institute of Health, USA). The results shown are n = 6 replicates for each group. The experiment was representative of two independent experiments.
(TIF)

Figure S4 **Bioassay of** ***CaMSRB2*** **-overexpressing rice plants treated with 10% PEG.** Four-week-old seedlings were cultured in MS medium with 10% polyethylene glycol (PEG) (6000) for two days. The fresh weight (FW) of the plants that were treated with 10% PEG was measured, and their percentage weight was presented by comparing their FW to that of non-treated plants. Similarly, the percentage of dry weight (DW) was presented by comparing the weights of the plants with/without the PEG treatment. WT: WT; 4, 8, 14, 23, 29, and 30: independent transgenic lines harboring the *MSRB2-Bar* vector. The results shown are the mean ± SD, n = 5 replicates for each group (***P<0.001, **P<0.01, and *P<0.05 compared to WT).
(TIF)

Figure S5 **Detection of proteins containing methionine sulfoxide (MetSO) residues by western blotting.** (A) The WT and CaMSRB2-transformed rice were treated under drought conditions for 2 days. Thereafter, equal amounts of the leaf protein extracts were loaded on a 10% SDS-PAGE gel. (B) Equal amounts of the leaf protein extracts were subjected to SDS-gel electrophoresis followed by western blot analysis using the methionine sulfoxide polyclonal antibody (Cayman). The band intensities were quantified with the Multi Gauge V2.3 program (Fujifilm). kDa, molecular mass indicators (in kDa). The experiment was representative of two independent experiments.
(TIF)

Figure S6 **Trypsin-digested fragments of PBGD.** The underlined sequences represent the five peptide fragments including methionine after trypsin treatment. The arrows show the position of eight methionine residues.
(TIF)

Figure S7 **Venn diagrams of differentially expressed genes.** Blue and yellow represent the genes that were down- (A) or up-regulated (B) more than 2-fold in *MSRB2*-mini and *MSRB2-Bar* plants that were grown under normal conditions compared to WT plants that were grown under normal conditions, respectively. A total of 27 and 73 genes were commonly down- and up-regulated in both the *MSRB2*-mini and *MSRB2-Bar* plants that were grown under normal conditions, respectively.
(TIF)

Figure S8 **MapMan metabolic overview of drought stress-responsive genes in both the WT and transgenic plants.** The boxes represent the log2 expression values of stress-responsive genes. The genes in red were up-regulated in response to stress, while the expression of those in blue was repressed.
(TIF)

Figure S9 **Tetrapyrrole pathway of drought stress-responsive genes.** (A) The tetrapyrrole pathway of drought stress-responsive genes in both the WT and transgenic plants. (B) The tetrapyrrole pathway of drought stress-responsive genes that were repressed only in the WT plants. The boxes represent the log2 expression values of stress-responsive genes. The genes in red were up-regulated in response to stress, while the expression of those in blue was repressed.
(TIF)

Figure S10 **Expression levels of OsMSRB3 and OsMSRB5 under drought stress treatment as determined by real-time PCR.** For drought stress, the water was removed from four-week-old plants, and these plants were incubated in the greenhouse for 2 days. The results shown are the mean ± SD, n = 3 replicates for each group. The experiment was representative of three independent experiments.
(TIF)

Table S1 **Primers that were used for PCR/real-time PCR.**
(PDF)

Table S2 **Mapping results of T-DNA flanking sequences by FSTVAL.**
(PDF)

Table S3 **Identified proteins using LC-MS/MS analysis.**
(PDF)

Table S4 **Lists of down- and up-regulated gene in the transgenic plants compared to the WT plants that were grown under normal conditions.**
(PDF)
