# Supplementary figures and images for: A Pepper MSRB2 Gene Confers Drought Tolerance in Rice through the Protection of Chloroplast-Targeted Genes
Source: PLoS One. 2014 Mar 10;9(3):e90588. doi: 10.1371/journal.pone.0090588 (PMC3948683; doi:10.1371/journal.pone.0090588)

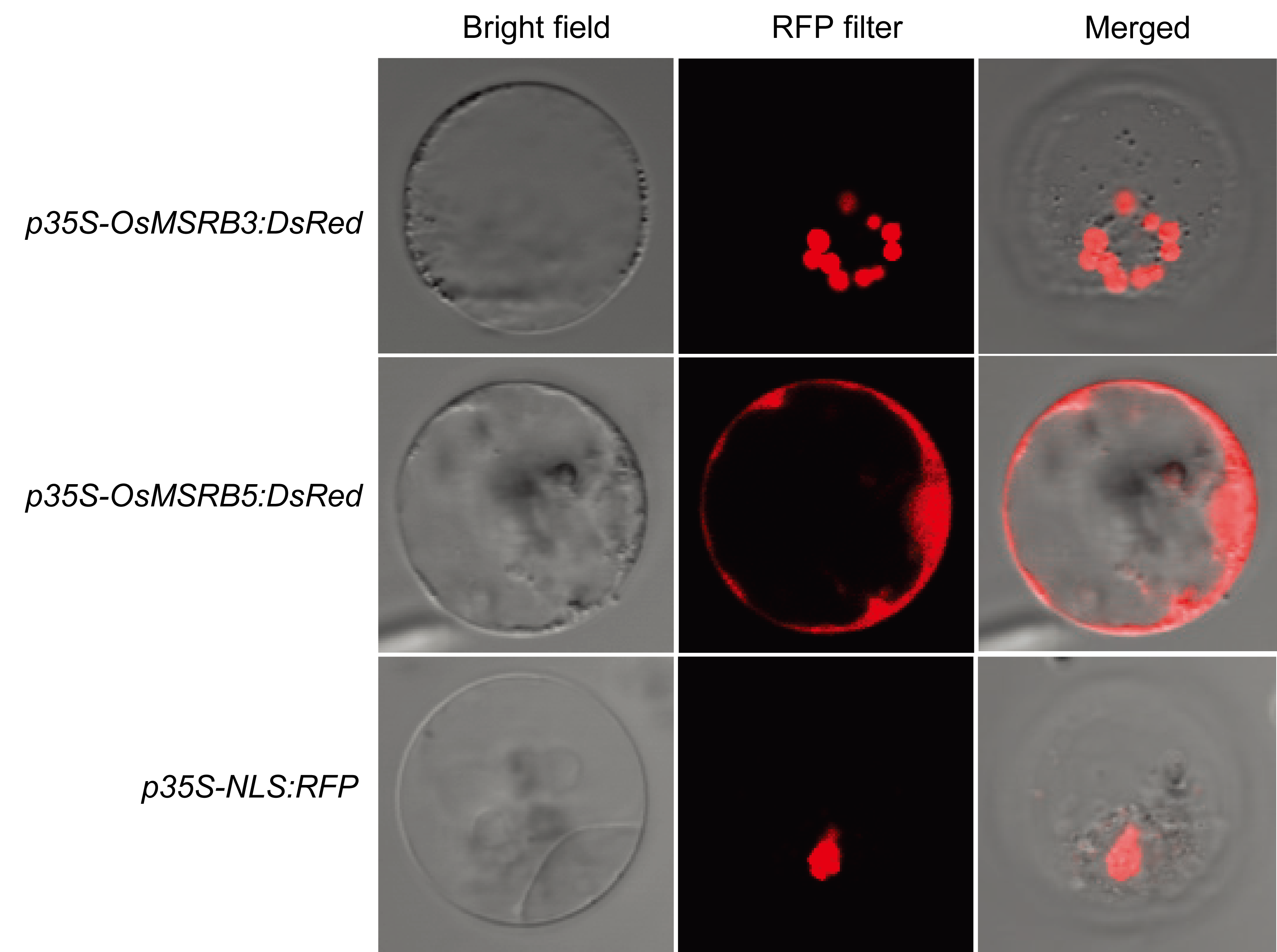

Supplement: Figure S1 — Subcellular Localization of CaMSRB3 and CaMSRB5 by transient expression. The expression of CaMSRB3 and CaMSRB5 are fused in-frame to the DsRed and their expression was driven by the CaMV 35S promoter in protoplasts of rice. The signals were examined under a confocal microscope. As a control for nucleus NLS::RFP were expressed in protoplasts of rice. These results showed OsMSRB3, OsMSRB5 are localized in chloroplast and cytosol, respectively. (TIF) [file pone.0090588.s001.tif]

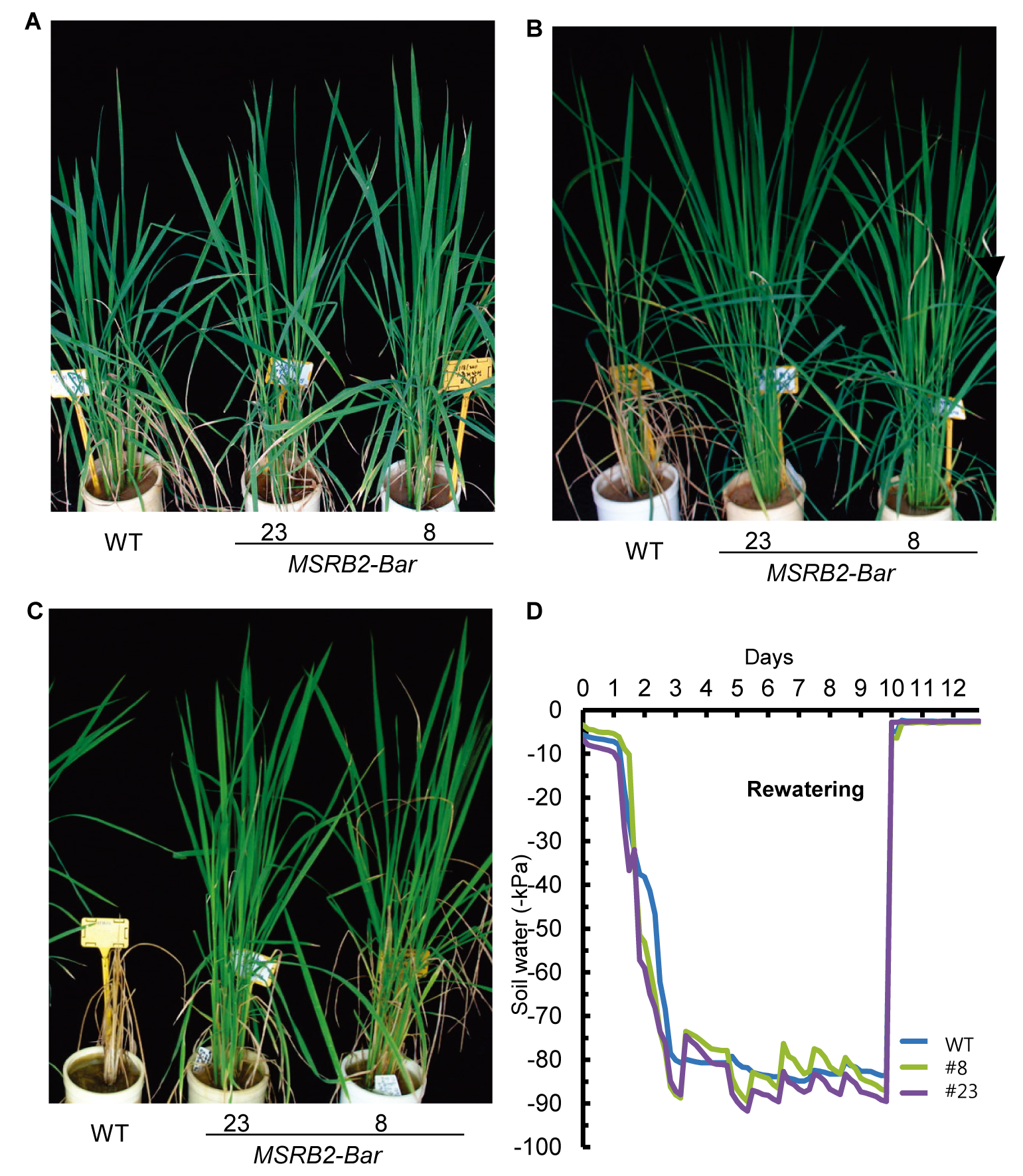

Supplement: Figure S2 — Growth of CaMSRB2-overexpressing rice plants upon drought treatment. (A) CaMSRB2-overexpressing lines (lines 8 and 23) and WT plants grown under normal conditions. (B) Twelve-week-old transgenic and WT plants that were not watered for 5 days. (C) Twelve-week-old transgenic and WT plants that were not watered for 7 days. (D) Measurement of soil water loss under drought stress. The soil moisture suction was recorded using a soil tensiometer (DIK-3023). The experiment was representative of three independent experiments. (TIF) [file pone.0090588.s002.tif]

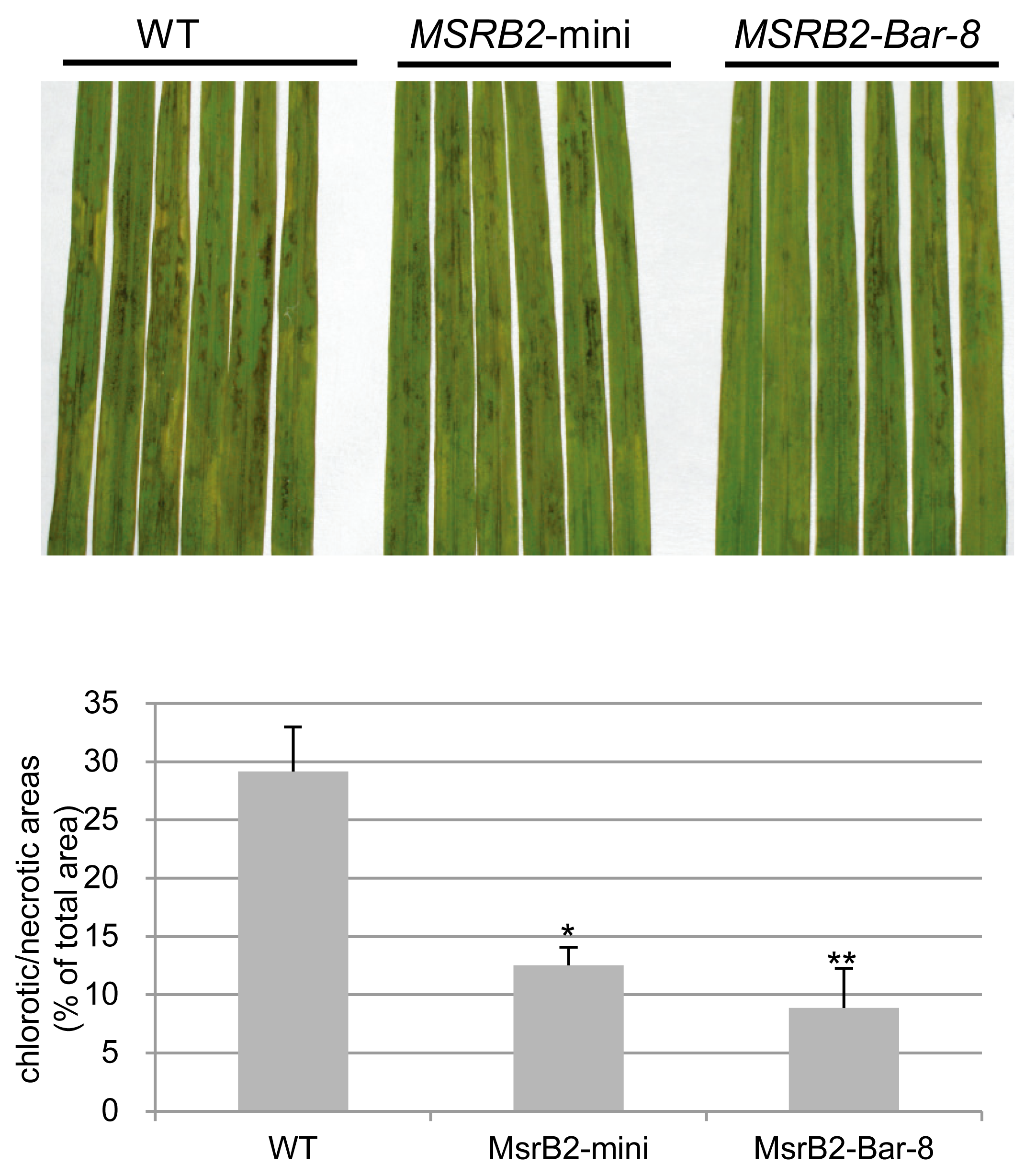

Supplement: Figure S3 — Response of CaMSRB2-transformed rice to an oxidative stress. The detached leaves of wild-type, MSRB2-Bar and MSRB2-mini plants were incubated in MS medium containing 0.1 mM methyl viologen (MV) for 50 h. The resistance of the leaves to oxidative stress was judged visually. And then we quantified the chlorotic/necrotic areas per total area (%) using Image J software (Wayne Rasband, National Institute of Health, USA). The results shown are n = 6 replicates for each group. The experiment was representative of two independent experiments. (TIF) [file pone.0090588.s003.tif]

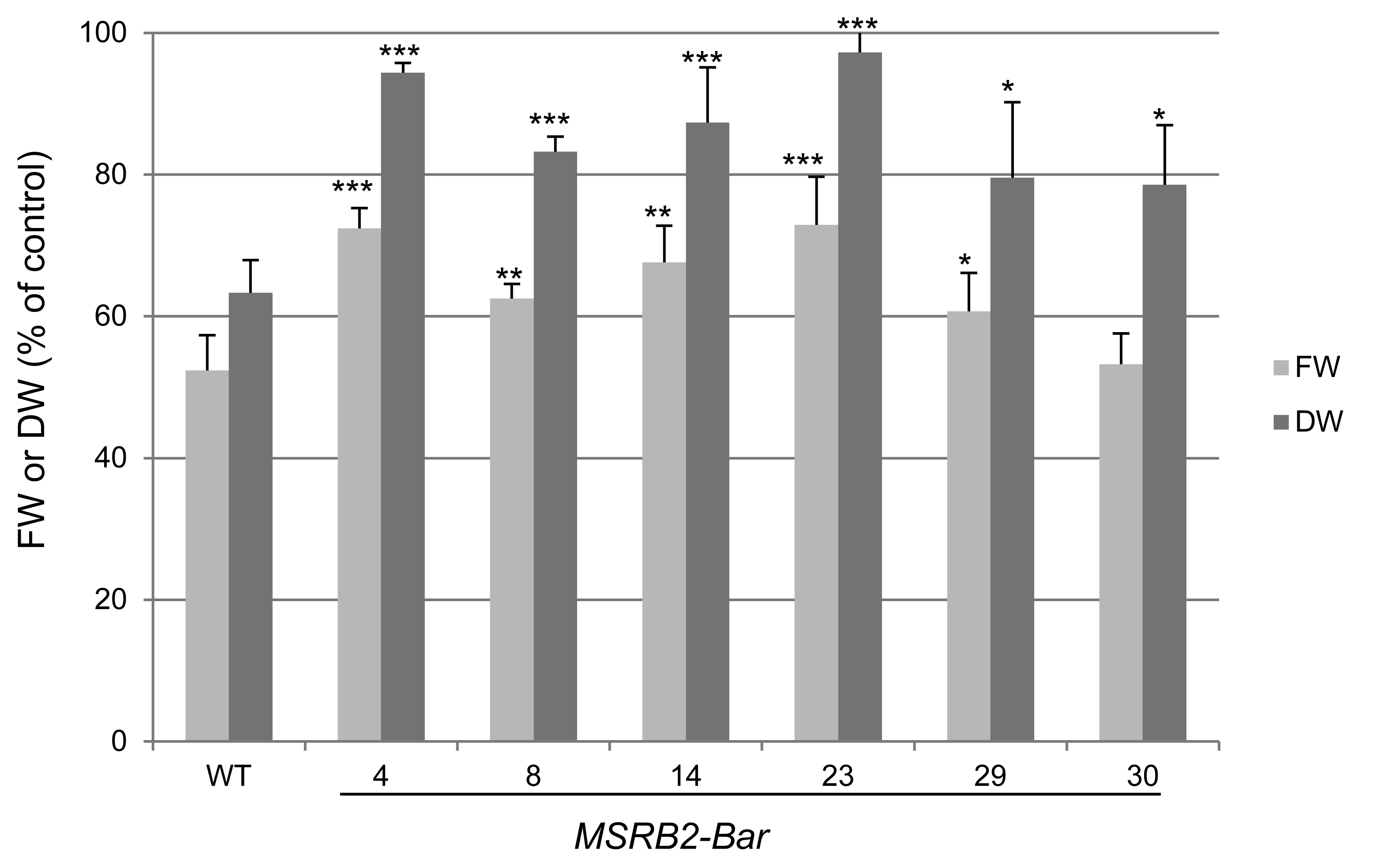

Supplement: Figure S4 — Bioassay of CaMSRB2 -overexpressing rice plants treated with 10% PEG. Four-week-old seedlings were cultured in MS medium with 10% polyethylene glycol (PEG) (6000) for two days. The fresh weight (FW) of the plants that were treated with 10% PEG was measured, and their percentage weight was presented by comparing their FW to that of non-treated plants. Similarly, the percentage of dry weight (DW) was presented by comparing the weights of the plants with/without the PEG treatment. WT: WT; 4, 8, 14, 23, 29, and 30: independent transgenic lines harboring the MSRB2-Bar vector. The results shown are the mean ± SD, n = 5 replicates for each group (***P<0.001, **P<0.01, and *P<0.05 compared to WT). (TIF) [file pone.0090588.s004.tif]

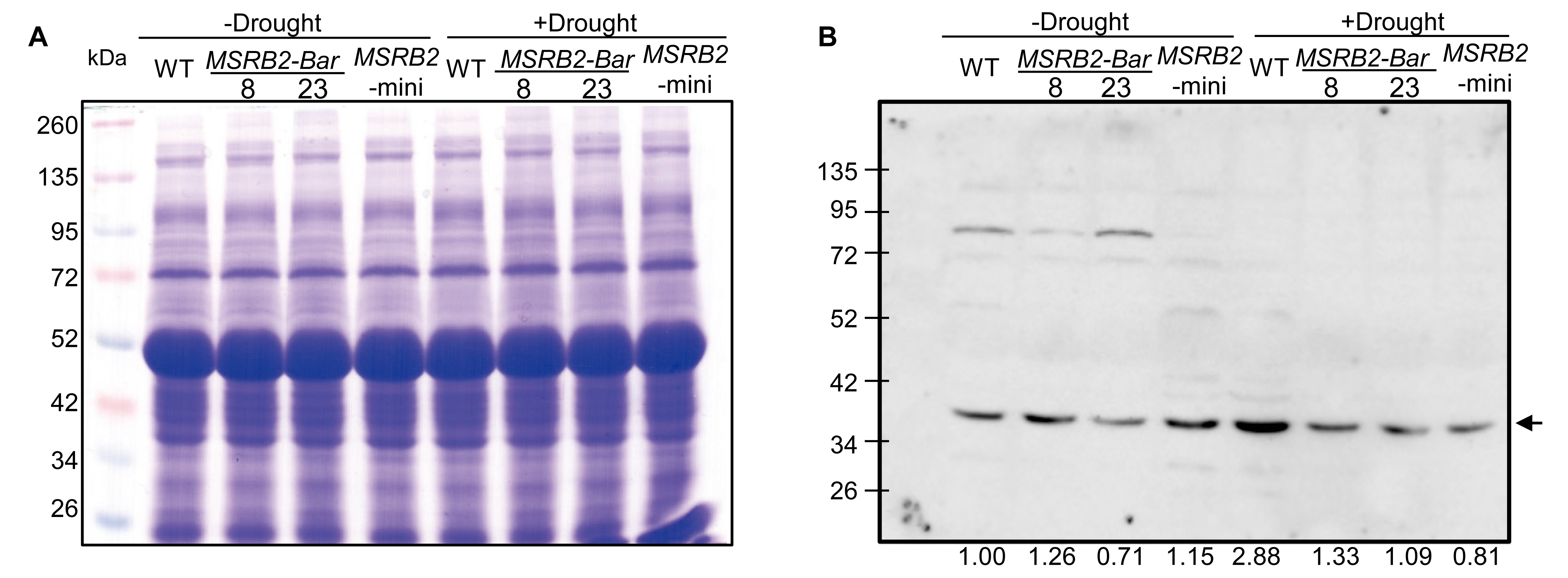

Supplement: Figure S5 — Detection of proteins containing methionine sulfoxide (MetSO) residues by western blotting. (A) The WT and CaMSRB2-transformed rice were treated under drought conditions for 2 days. Thereafter, equal amounts of the leaf protein extracts were loaded on a 10% SDS-PAGE gel. (B) Equal amounts of the leaf protein extracts were subjected to SDS-gel electrophoresis followed by western blot analysis using the methionine sulfoxide polyclonal antibody (Cayman). The band intensities were quantified with the Multi Gauge V2.3 program (Fujifilm). kDa, molecular mass indicators (in kDa). The experiment was representative of two independent experiments. (TIF) [file pone.0090588.s005.tif]

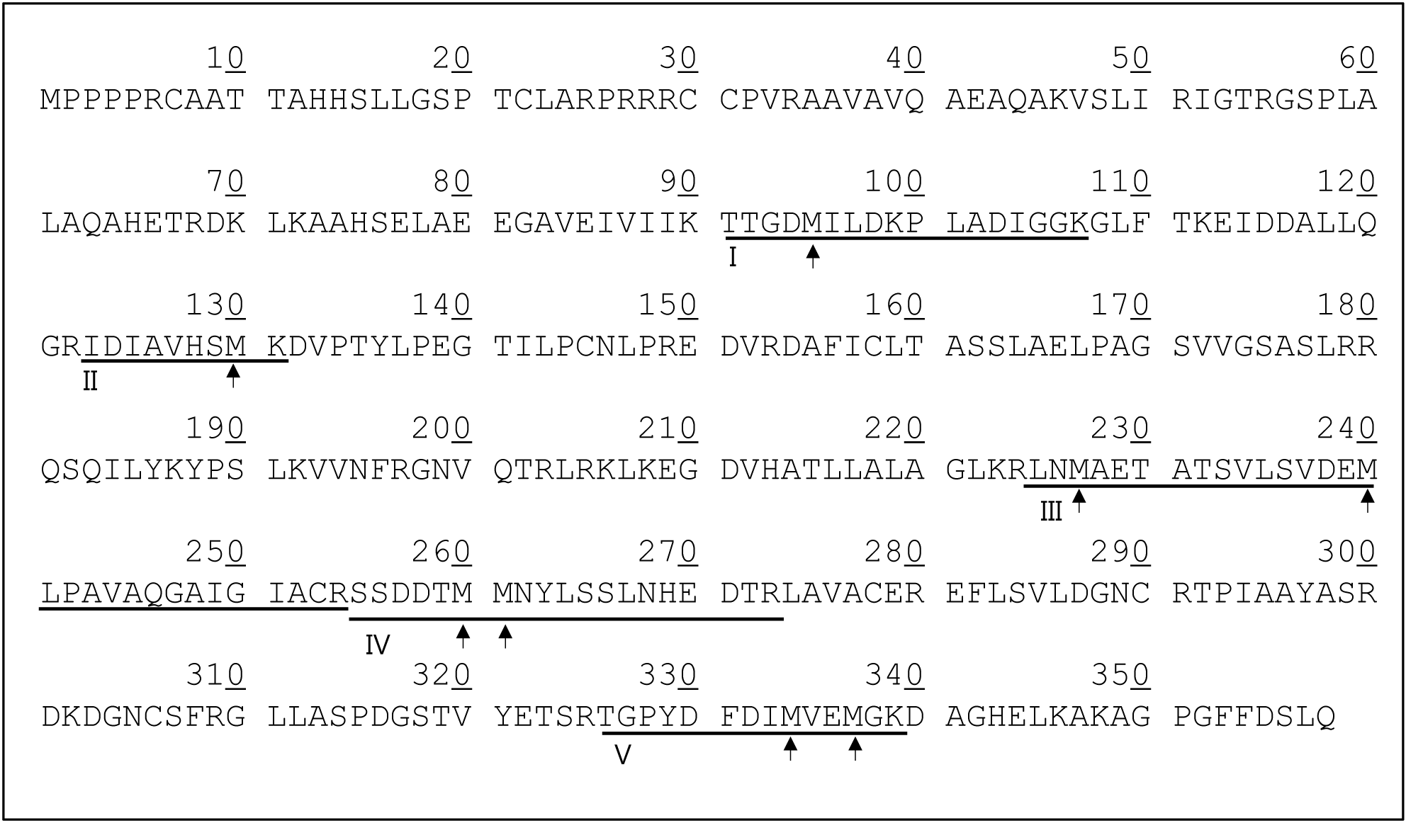

Supplement: Figure S6 — Trypsin-digested fragments of PBGD. The underlined sequences represent the five peptide fragments including methionine after trypsin treatment. The arrows show the position of eight methionine residues. (TIF) [file pone.0090588.s006.tif]

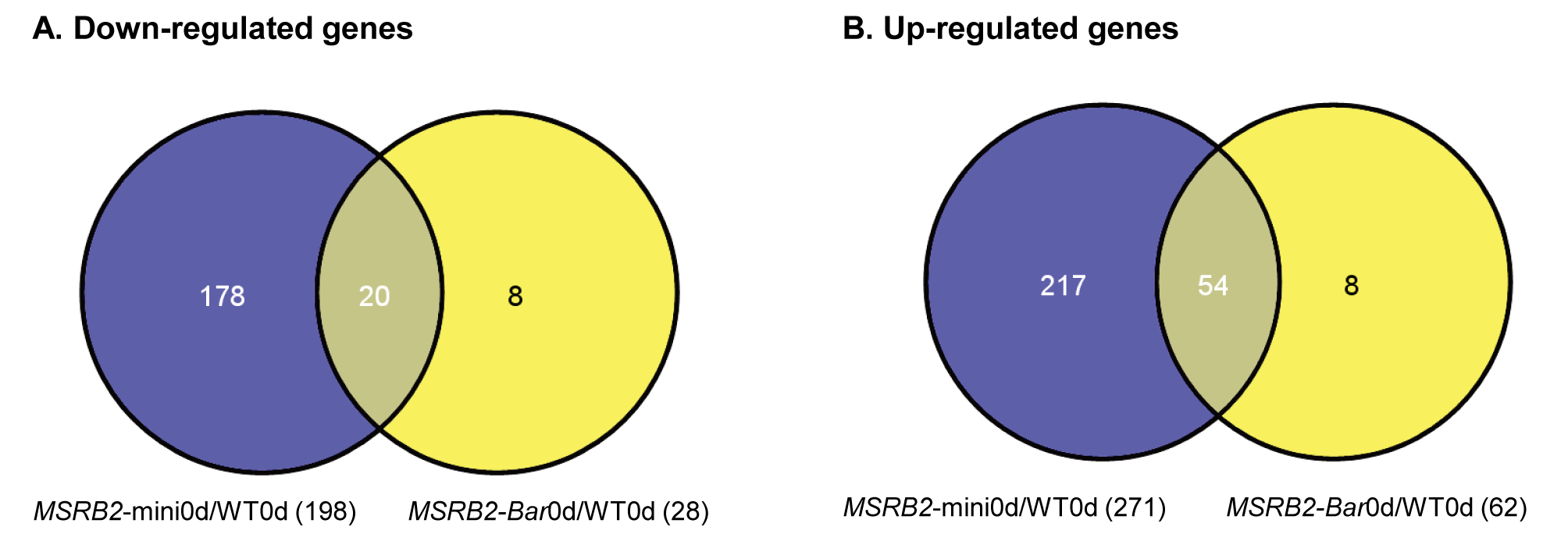

Supplement: Figure S7 — Venn diagrams of differentially expressed genes. Blue and yellow represent the genes that were down- (A) or up-regulated (B) more than 2-fold in MSRB2-mini and MSRB2-Bar plants that were grown under normal conditions compared to WT plants that were grown under normal conditions, respectively. A total of 27 and 73 genes were commonly down- and up-regulated in both the MSRB2-mini and MSRB2-Bar plants that were grown under normal conditions, respectively. (TIF) [file pone.0090588.s007.tif]

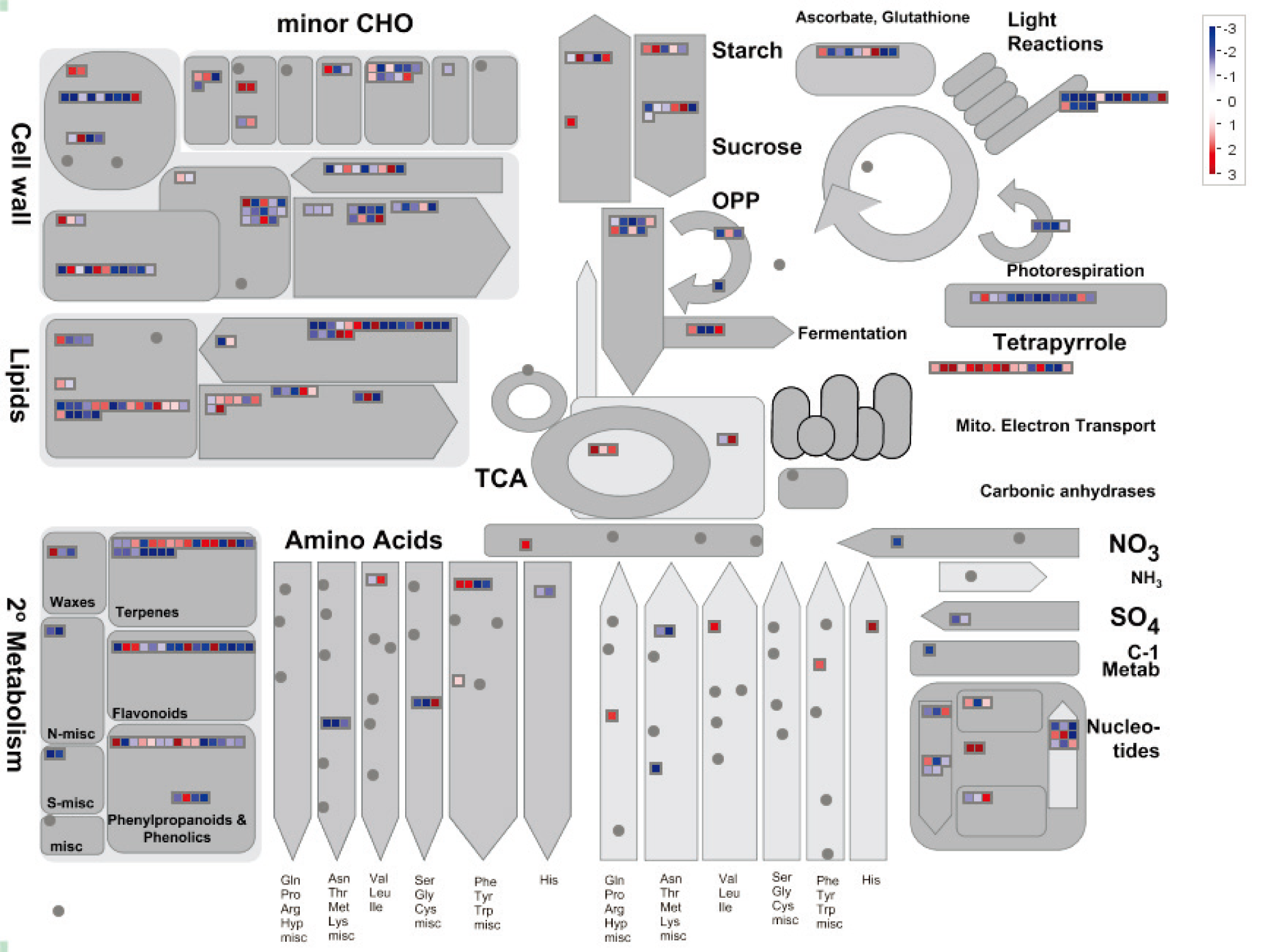

Supplement: Figure S8 — MapMan metabolic overview of drought stress-responsive genes in both the WT and transgenic plants. The boxes represent the log2 expression values of stress-responsive genes. The genes in red were up-regulated in response to stress, while the expression of those in blue was repressed. (TIF) [file pone.0090588.s008.tif]

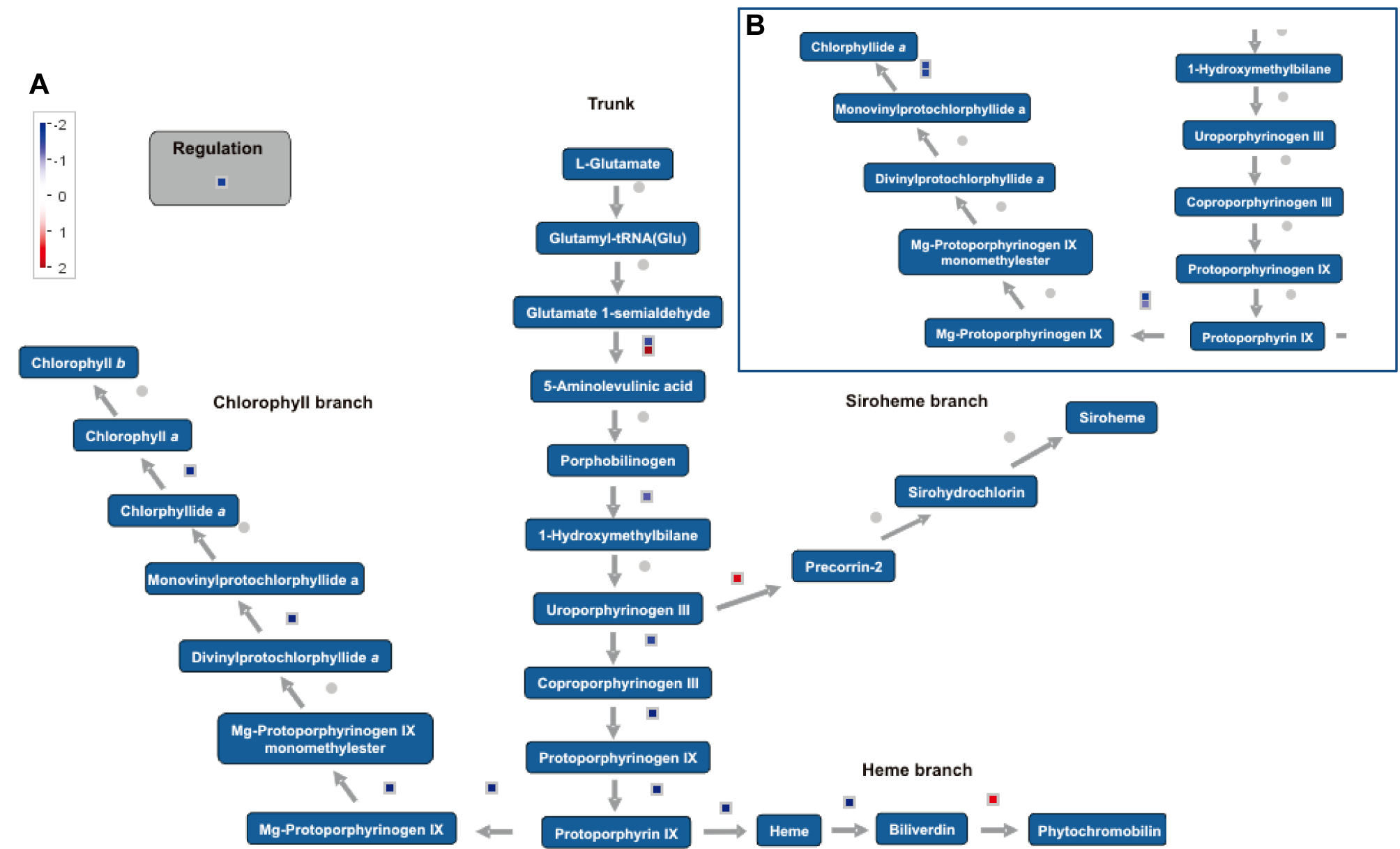

Supplement: Figure S9 — Tetrapyrrole pathway of drought stress-responsive genes. (A) The tetrapyrrole pathway of drought stress-responsive genes in both the WT and transgenic plants. (B) The tetrapyrrole pathway of drought stress-responsive genes that were repressed only in the WT plants. The boxes represent the log2 expression values of stress-responsive genes. The genes in red were up-regulated in response to stress, while the expression of those in blue was repressed. (TIF) [file pone.0090588.s009.tif]

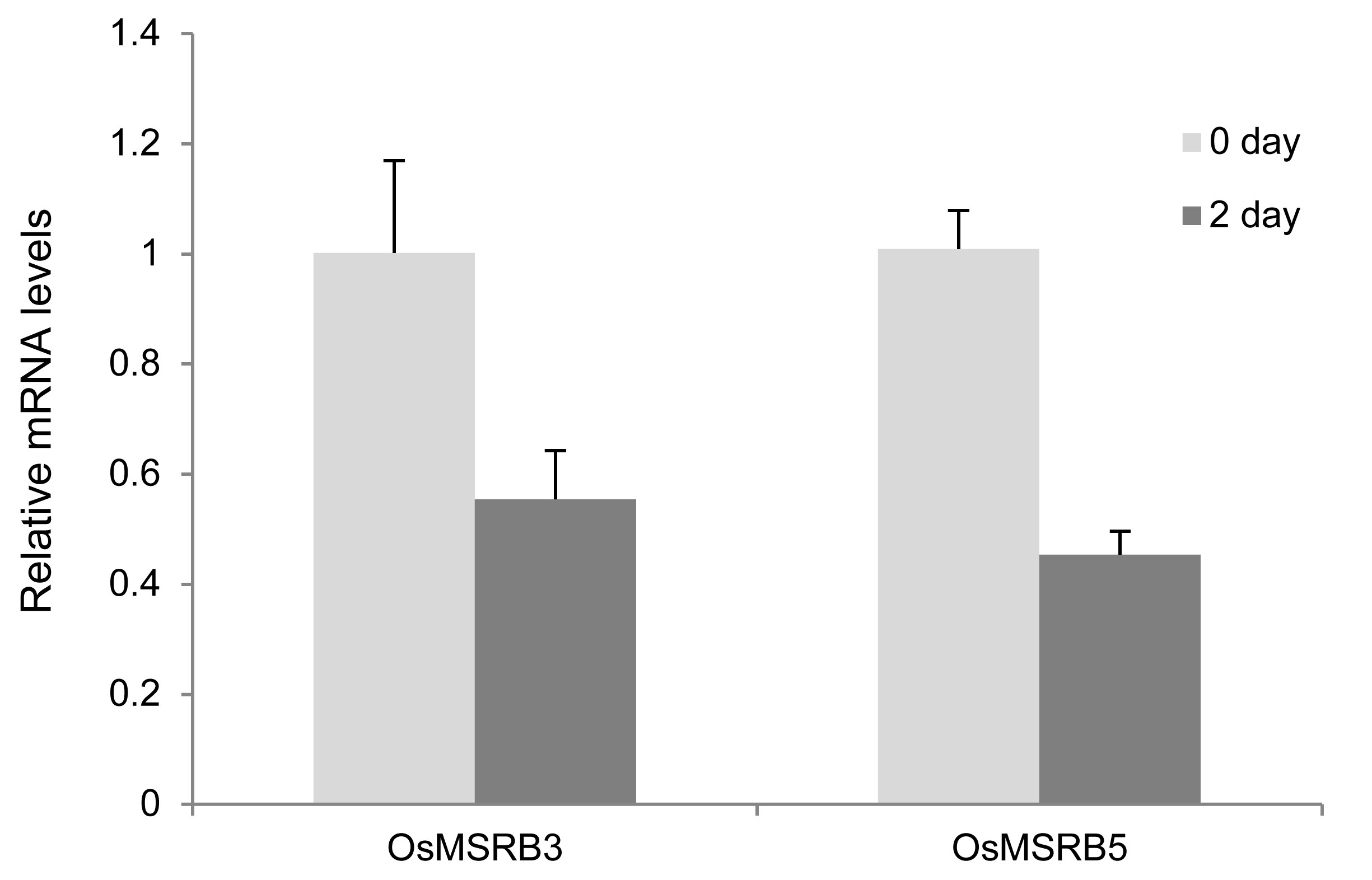

Supplement: Figure S10 — Expression levels of OsMSRB3 and OsMSRB5 under drought stress treatment as determined by real-time PCR. For drought stress, the water was removed from four-week-old plants, and these plants were incubated in the greenhouse for 2 days. The results shown are the mean ± SD, n = 3 replicates for each group. The experiment was representative of three independent experiments. (TIF) [file pone.0090588.s010.tif]
