# Supplementary material for: A Pepper MSRB2 Gene Confers Drought Tolerance in Rice through the Protection of Chloroplast-Targeted Genes
Source: PLoS One. 2014 Mar 10;9(3):e90588. doi: 10.1371/journal.pone.0090588 (PMC3948683; doi:10.1371/journal.pone.0090588)
Supplement: Table S1 — Primers that were used for PCR/real-time PCR. (PDF) [file pone.0090588.s011.pdf]

Table S1. Primers that were used for PCR/real-time PCR

Primer ID	Sequence	Product (bps)
OsMSRB3_XbF	GGGTCTAGAAAAATGGGCGTCCAGCATCT	705
OsMSRB3-TGA-NR	TTTGCGGCCGCCAAGCTTACTATCTTCTGAGGC	
OsMSRB5_XbF	GGGTCTAGAATGGCGTCGTCGGGGGAC	423
OsMSRB5-TGA-NR	TTGCGGCCGCCGGAGGCCGGGGTGAACTTG	
CaMSR2-Xb-F	GGGTCTAGAATGGGTTCTCAGATTCTCAAA	570
CaMSR2-TGA-NR	TTTGCGGCCGCCGGAATTTGCCGGTGTAAAC	
CaMSRB2_R411	GTCAGGAGTGCGATTAATAGC	201
CaMSRB2_F230	CCTGAGCAGTTCCGTATTCT	
CaMSRB2_B1	AAAAAGCAGGCTGCGACTTTGAATTGGTCTTTG	882
CaMSRB2_B2	AGAAAGCTGGGTATATCAAACTTTGTCTAGATTATC	
BAR280_F	GAGTCGACCGTGTACGTCTC	291
BAR550_R	GTCAAATCTCGGTGACGGG	
rab21_F	ACCACCGACACCGGCGAGAA	160
rab21_R	TTGCGCTCGGCTAGCTCATC	
RbcS_F	CACTCACCGTGGAGGATCTT	350
RbcS_R	AGCGCGATGCTTGATCTTAG	
OsMSRB3-564R*	CGCACAGGTGATCTCTATTC	157
OsMSRB3-408F*	GGGTGTTTATGAATGTGCTG	
OsMSRB5-360R*	ATCAGTAGGCGTCTTGAAGC	174
OsMSRB5-187F*	TCCACAACCAAGTTTGATTC	
Os01g0938100_F*	GAGGGCGTGCTCCAGTCGGT	223
Os01g0938100_R*	GCACACAGTACATGCTCAAGGCAGT	
Os04g0414700_F*	GCGCTCGACTCGCCGTTCTG	231
Os04g0414700_R*	ACAACAGCGTACATCCGCAAGAGG	
Os08g0502700_F*	GGGTGCCCTGAGTGGAGTGGA	206
Os08g0502700_R*	TGCCGCTGTTCCGTTCTGCC	
Os03g0778100_F*	GCGAGAAGAAGCCGGCCATGA	210
Os03g0778100_R*	AGCCCTCTTCCTCCACTGCCA	
Os04g0644600_F*	TTGCATGGGGGATTTCTGAT	205
Os04g0644600_R*	TACCTGTGACTTGTACAGGA	
Os08g0248800_F*	CAAACACGCATTCAAAAAGA	183
Os08g0248800_R*	ATATGCAGCTCTCGGATCAC	
Os07g0693800_F*	GACAGTGGACAGGGACTATG	228
Os07g0693800_R*	CTGACATAGTGGTCGCGTT	
Os02g0596000_F*	TGAAAGAAGGGCTGGGCTAC	215
Os02g0596000_R*	ACATTACGGAGCCGCAACA	
Os03g0736400_F*	AACACCGCCATATCTTGAGG	208
Os03g0736400_R*	CTTCTTCTCAGCCCAGGTTG	
Os04g0465500_F*	TTGACTTCCCGTACATTTGC	202
Os04g0465500_R*	CTGTCAGTCTGGACAATGTT	
tubulin_F*	ACCGTGATTGATGAGGTGAG	205
tubulin_R *	CAGCGTTGAACACAAGGAAG	
Os06g0107700_stu_F	ccaggcctATGGCCGCCGTCACG	1086
Os06g0107700_N_R	ttgcggccgccGTAGACTTCCACGTTCCAT	
Os11g0707000_Rv_F	gatatcATGGCTGCTGCCTTCTC	1299
Os11g0707000_N_R	aagcggccgccGCTGGATGGCGCAGAAC	
Os12g0625000_stu_F	ccaggcctatgTCCACGAGCCGCCATTTC	1041
Os12g0625000_N_R	tgcggccgccTTCAACCACCATGTTTTCAG	
Os02g0168800_stu_F	ccaggcct TCTTCCCCGGAGATGCC	1074
Os02g0168800_N_R	tTgcggccgccTTGCAAGCTATCAAAGAAG	
Os02g0436400_stu_F	ccaggcct ATGCTCGCCTCCACCTTTG	1029
Os02g0436400_N_R	tgcggccgccTCTCATGTTGCCCTCTCTTA	
