# Supplementary material for: A Pepper MSRB2 Gene Confers Drought Tolerance in Rice through the Protection of Chloroplast-Targeted Genes
Source: PLoS One. 2014 Mar 10;9(3):e90588. doi: 10.1371/journal.pone.0090588 (PMC3948683; doi:10.1371/journal.pone.0090588)
Supplement: Table S2 — Mapping results of T-DNA flanking sequences by FSTVAL. (PDF) [file pone.0090588.s012.pdf]

Table S2. Mapping results of T-DNA flanking sequences by FSTVAL

Query Name	Read length	A/NA	T-DNA end	Adaptor start	matching length	query start	query end	Chr.	chr start	chr end	type	copies
MSRB2-Bar-1-R1	623	A	72	600	299	301	599	10	15728411	15728709	650bp at 5nd of Os10g0429500	1
MSRB2-Bar-1-L1	620	Vector	18	597								
MSRB2-Bar-3-R1	188	Vector	71	155								2
MSRB2-Bar-3-R2	126	NA	72	-1								
MSRB2-Bar-3-L1	502	A	-1	469	299	170	468	11	17171622	17171324		
MSRB2-Bar-3-L2	278	A	-1	249	248	1	248	4	21629644	21629891		
MSRB2-Bar-4-R1	256	A	40	223	161	62	222	2	33365046	33365206	intergenic region	1
MSRB2-Bar-4-L1	586	A	28	564	538	26	563	2	33365042	33364505		
MSRB2-Bar-5-R1	345	A	69	312	244	68	311	11	17170810	17170567		3
MSRB2-Bar-5-R2	213	NA	67	-1								
MSRB2-Bar-5-L1	81	NA	-1	-1								
MSRB2-Bar-5-L2	415	A	-1	395								
MSRB2-Bar-5-L3	220	A	-1	188								
MSRB2-Bar-5-L4	126	A	-1	97	96	1	96	12	787461	787556		
MSRB2-Bar-6-R1	446	A	67	427	362	65	426	11	20076386	20076025		2
MSRB2-Bar-6-R2	180	Vector	69	147								
MSRB2-Bar-6-L1	484	A	-1	465	299	166	464	11	17171622	17171324		
MSRB2-Bar-6-L2	0	NA	-1	-1								
MSRB2-Bar-6-L3	146	A	-1	113								
MSRB2-Bar-7-R1	148	Vector	75	119								2
MSRB2-Bar-7-L1	581	A	14	552								
MSRB2-Bar-7-L2	248	A	14	216	207	9	215	11	27175490	27175696		
MSRB2-Bar-8-R1	836	A	29	-1	601	29	629	1	41711122	41711722		1
MSRB2-Bar-8-L1	472	A	-1	-1	333	140	472	1	41713700	41713368	intergenic region	
MSRB2-Bar-9-R1	61	NA	48	-1								3
MSRB2-Bar-9-R2	606	A	45	-1	564	43	606	4	20708627	20709188		
MSRB2-Bar-9-L1	614	A	18	597								
MSRB2-Bar-9-L2	447	A	-1	427	299	128	426	11	17171622	17171324		
MSRB2-Bar-9-L3	221	A	-1	189	189	1	188	4	20708587	20708399		
MSRB2-Bar-12-R1	39	NA	35	-1								3
MSRB2-Bar-12-L1	751	A	-1	-1	736	16	751	9	12830881	12830146		
MSRB2-Bar-12-L2	621	A	-1	602	581	21	601	9	12785169	12785749		
MSRB2-Bar-12-L3	85	NA	-1	-1								
MSRB2-Bar-12-L4	161	A	23	132								
MSRB2-Bar-13-L1	260	A	-1	228	227	1	227	1	10115418	10115192		2
MSRB2-Bar-13-L2	201	A	-1	176	144	32	175	1	10115500	10115643		
MSRB2-Bar-14-R1	686	A	68	-1	593	90	682	1	25070441	25069849	intergenic region	1
MSRB2-Bar-14-L1	286	A	-1	254	250	9	258	1	25070464	25070713		
MSRB2-Bar-15-R1	264	Vector	69	189								1
MSRB2-Bar-15-L1	531	A	50	512	465	47	511	11	2045597	2045133	5' UTR of J023012M05	
MSRB2-Bar-17-R1	187	Vector	69	154								1
MSRB2-Bar-17-L1	482	A	-1	462	299	163	461	11	17171622	17171324		
MSRB2-Bar-18-R1	210	NA	64	-1								1
MSRB2-Bar-22-R1	509	A	68	-1	441	69	509	2	28088186	28088626	intergenic region	1
MSRB2-Bar-23-L1	721	A	-1	701	696	5	700	8	2470962	2470267	intergenic region	1
MSRB2-Bar-25-R1	106	NA	71	-1								1
MSRB2-Bar-29-R1	242	A	72	209	137	72	208	6	27732321	27732185	6th exon of Os06g0655100	1
MSRB2-Bar-29-L1	222	A	-1	202	201	1	201	6	27731846	27731646		
MSRB2-Bar-30-R1	278	A	70	259	104	155	258	2	724370	724267	intergenic region	1
MSRB2-Bar-30-L1	674	A	-1	-1	424	1	424	chr.02	724532	724955		
MSRB2-mini-1-L1	373	A	-1	334	329	5	333	9	22170328	22170655	Intergenic	1
MSRB2-mini-2-R1	195	Vector	59	-1								1
MSRB2-mini-3-R1	400	A	61	-1	235	158	392	2	24459205	24458971	Intergenic	1
MSRB2-mini-4-R1	297	A	71	-1	197	100	296	2	19779018	19778822	Intergenic	1
MSRB2-mini-5-R1	439	Vector	71	411								1
