# Supplementary material for: A Pepper MSRB2 Gene Confers Drought Tolerance in Rice through the Protection of Chloroplast-Targeted Genes
Source: PLoS One. 2014 Mar 10;9(3):e90588. doi: 10.1371/journal.pone.0090588 (PMC3948683; doi:10.1371/journal.pone.0090588)
Supplement: Table S3 — Identified proteins using LC-MS/MS analysis. (PDF) [file pone.0090588.s013.pdf]

Table S3. Identified proteins using LC-MS/MS analysis.

Identified Proteins	Accession Number		Molecular Weight	WT		*MSRB2-Ba* r-8	
				0 Day	2Day	MsrB2-B	M82D
**Ribulose bisphosphate carboxylase/oxygenase activase, chloroplastic**	RCA_ORYSJ	**Os11g0707000**	51 kDa	38.1	65.44	97.47	97.84
Fructose-bisphosphate aldolase, chloroplastic	ALFC_ORYSJ	Os11g0171300	42 kDa	142.31	119.97	113.71	125.94
Serpin-ZXA	SPZXA_ORYSJ	Os03g0610650	42 kDa	1.12	0	7.64	5.2
Probable protein phosphatase 2C	P2C05_ORYSJ	Os01g0552300	42 kDa	0	0	0	2.08
Actin-1	ACT1_ORYSI	Os03g0718100	42 kDa	2.24	2.73	2.87	0
Ferredoxin--NADP reductase, embryo isozyme, chloroplastic	FENR3_ORYSJ	Os07g0147900	42 kDa	2.24	0	0	0
UDP-arabinopyranose mutase 1	RGP1_ORYSJ	Os03g0599800	41 kDa	0	4.54	4.78	3.12
Alcohol dehydrogenase class-3	ADHX_ORYSJ	Os02g0815500	41 kDa	2.24	4.54	0.96	2.08
Putative 12-oxophytodienoate reductase 11	OPR11_ORYSJ	Os04g0542200	41 kDa	0	2.73	0	0
Quercetin 3-O-methyltransferase 1	OMT1_ORYSJ	Os08g0157500	**40 kDa**	7.84	10	5.73	10.41
Peroxisomal (S)-2-hydroxy-acid oxidase GLO4	GLO4_ORYSI		**40 kDa**	1.12	0.91	0.96	3.12
Actin-related protein 7	ARP7_ORYSI		**40 kDa**	0	0	1.91	2.08
Protein disulfide isomerase-like 2-1	PDI21_ORYSJ	s05g0156300	**40 kDa**	0	1.82	0.96	2.08
**Ferredoxin--NADP reductase, leaf isozyme, chloroplastic**	FENR1_ORYSJ	Os06g0107700	**40 kDa**	15.69	6.36	2.87	3.12
Peroxisomal (S)-2-hydroxy-acid oxidase GLO5	GLO5_ORYSI	Os07g0152900	**40 kDa**	34.74	35.45	33.44	30.18
Peroxisomal (S)-2-hydroxy-acid oxidase GLO1	GLO1_ORYSI	Os03g0786100	**40 kDa**	34.74	46.35	47.78	40.59
Probable cinnamyl alcohol dehydrogenase 1	CADH1_ORYSJ	Os10g0194200	**39 kDa**	1.12	5.45	2.87	6.25
Fructose-bisphosphate aldolase cytoplasmic isozyme	ALF_ORYSJ	Os05t0402700	**39 kDa**	20.17	28.17	28.67	18.74
Methylthioribose-1-phosphate isomerase	MTNA_ORYSI	Os11g0216900	**39 kDa**	3.36	8.18	5.73	4.16
Probable UDP-arabinopyranose mutase 2	RGP2_ORYSJ	Os04g0660400	**39 kDa**	2.24	4.54	0.96	2.08
Glutamine synthetase cytosolic isozyme 1-1	GLN11_ORYSJ	Os02g0735200	**39 kDa**	1.12	3.64	3.82	3.12
Cycloartenol-C-24-methyltransferase 1	SMT1_ORYSJ	Os07g0206700	**39 kDa**	0	0	1.91	0
**Porphobilinogen deaminase, chloroplastic**	HEM3_ORYSJ	**Os02g0168800**	**38 kDa**	12.33	4.54	5.73	15.61
Diaminopimelate epimerase, chloroplastic	DAPF_ORYSJ	Os12g0567200	**38 kDa**	8.96	1.82	1.91	0
**Probable dihydrodipicolinate reductase 1, chloroplastic**	DAPB1_ORYSJ	Os02g0436400	**37 kDa**	2.24	0.91	5.73	1.04
Fructose-1,6-bisphosphatase, cytosolic	F16P2_ORYSI	Os01g0866400	**37 kDa**	11.21	8.18	5.73	12.49
Malate dehydrogenase, glyoxysomal	MDHG_ORYSJ	Os12g0632700	**37 kDa**	3.36	3.64	0.96	5.2
Electron transfer flavoprotein subunit alpha, mitochondrial	ETFA_ORYSI	Os03g0835400	**37 kDa**	3.36	0	0	0
Glyceraldehyde-3-phosphate dehydrogenase, cytosolic	G3PC_ORYSI	Os08g0126300	**36 kDa**	22.41	19.09	25.8	19.78
Fructokinase-2	SCRK2_ORYSI	Os08g0113100	**36 kDa**	13.45	10.91	8.6	15.61
Probable voltage-gated potassium channel subunit beta	KCAB_ORYSJ	Os02g0817500	**36 kDa**	3.36	2.73	0.96	7.29
Ribose-phosphate pyrophosphokinase 4	KPRS4_ORYSJ	Os02g0714600	**36 kDa**	0	0.91	1.91	1.04
Serine/threonine-protein phosphatase PP1	PP1_ORYSJ	Os03g0268000	**36 kDa**	0	0	0	3.12
Malate dehydrogenase, cytoplasmic	MDHC_ORYSJ	Os10g0478200	**36 kDa**	16.81	23.63	21.98	21.86
Guanine nucleotide-binding protein subunit beta-like protein A	GBLPA_ORYSJ	Os01g0686800	**36 kDa**	16.81	9.09	7.64	7.29
Probable serine acetyltransferase 5	SAT5_ORYSJ	Os05g0533500	**36 kDa**	4.48	0.91	0	0
Guanine nucleotide-binding protein subunit beta-like protein B	GBLPB_ORYSJ	Os05g0552300	**36 kDa**	6.72	2.73	3.82	2.08
Fructokinase-1	SCRK1_ORYSI	Os01g0894300	**35 kDa**	11.21	6.36	15.29	7.29
Probable uridine nucleosidase 1	URH1_ORYSJ	Os08g0557900	**35 kDa**	3.36	1.82	1.91	2.08
60S ribosomal protein L5-2	RL52_ORYSJ	Os01g0896700	**35 kDa**	6.72	10.91	6.69	10.41
Probable protein phosphatase 2C	P2C01_ORYSJ	Os01g0164600	**35 kDa**	3.36	1.82	0.96	0
Homeobox protein knotted-1-like 11	KNOSB_ORYSJ	Os06g0646600	**35 kDa**	4.48	0	0.96	0
**Cysteine synthase**	CYSK1_ORYSJ	**Os12g0625000**	34 kDa	3.36	1.82	0	2.08
Probable succinyl-CoA ligase [ADP-forming] subunit alpha, mitochondrial	SUCA_ORYSJ	Os07g0577700	34 kDa	15.69	0.91	4.78	4.16
Probable pyridoxal biosynthesis protein PDX1.1	PDX11_ORYSJ	Os07g0100200	34 kDa	2.24	2.73	2.87	8.33
60S acidic ribosomal protein P0	RLA0_ORYSJ	Os08g0130500	34 kDa	8.96	10.91	9.56	6.25
PHD finger protein ALFIN-LIKE 9	ALFL9_ORYSI		30 kDa	0	2.73	0.96	3.12
Unknown protein DS12 from 2D-PAGE of leaf, chloroplastic	UP12_ORYSI	Os08g0242700	30 kDa	5.6	10	6.69	9.37
