# Supplementary material for: A Pepper MSRB2 Gene Confers Drought Tolerance in Rice through the Protection of Chloroplast-Targeted Genes
Source: PLoS One. 2014 Mar 10;9(3):e90588. doi: 10.1371/journal.pone.0090588 (PMC3948683; doi:10.1371/journal.pone.0090588)
Supplement: Table S4 — Lists of down- and up-regulated gene in the transgenic plants compared to the WT plants that were grown under normal conditions. (PDF) [file pone.0090588.s014.pdf]

Table S4. Lists of down- and up-regulated gene in the transgenic plants compared to the WT plants that were grown under normal conditions

Rab2 ID	mini0d /WT0d	Bar0d /WT0d	Callus /leaf	Description
Os04g0301500	-1.38	-1.20	**-5.50**	Basic helix-loop-helix dimerisation region bHLH domain containing protein.
Os02g0632800	-1.16	-1.24	**-5.31**	Protein kinase-like domain containing protein.
Os10g0118200	-1.64	-1.22	**-4.94**	Similar to O-methyltransferase ZRP4 (OMT).
Os01g0117200	-1.99	-1.15	**-4.49**	Similar to ARK protein (Fragment).
Os10g0118000	-1.61	-1.10	**-4.23**	Similar to O-methyltransferase ZRP4 (OMT).
Os11g0126500	-5.21	-4.69	**-3.14**	Plant MuDR transposase domain containing protein.
Os02g0269600	-1.13	-1.10	**-3.12**	Similar to Subtilase.
Os01g0564300	-1.55	-1.48	**-3.07**	Peptidylprolyl isomerase, FKBP-type domain containing protein.
Os03g0197200	-1.20	-1.60	**-2.95**	Similar to Sorbitol transporter.
Os07g0131100	-2.60	-2.26	**-2.65**	Legume lectin, beta domain containing protein.
Os04g0366000	-1.46	-1.03	**-2.39**	EGF domain containing protein.
Os02g0282000	-1.11	-1.06	**-1.61**	Disease resistance protein family protein.
AK065444	-1.74	-1.02	**-1.42**	NA
Os02g0570400	-1.41	-1.36	**-1.29**	Similar to Ent-kaurene synthase 1A.
Os12g0555200	-1.01	-1.29	-0.81	Similar to Probenazole-inducible protein PBZ1.
Os12g0221250	-1.83	-2.08	-0.48	Hypothetical protein.
Os09g0262000	-1.87	-2.36	-0.15	Similar to Cinnamoyl-CoA reductase.
Os02g0230300	-1.76	-1.24	0.35	Proline-and threonine-rich protein.
Os03g0197300	-2.30	-1.84	3.50	Cupin, RmlC-type domain containing protein.
Os01g0822900	-1.62	-1.58	4.52	Similar to Lipid transfer protein.
Os03g0226200	3.03	3.31	**4.89**	Non-symbiotic hemoglobin 2 (rHb2) (ORYsa GLB1b).
Os04g0206600	4.30	1.12	**4.63**	UDP-glucuronosyl/UDP-glucosyltransferase family protein.
Os03g0432100	2.11	1.16	**4.25**	Similar to Orthophosphate dikinase precursor.
Os04g0115200	3.85	3.43	**3.82**	Conserved hypothetical protein.
Os10g0419400	3.59	1.73	**3.70**	Similar to SIPL.
Os03g0115800	6.86	6.77	**3.55**	Conserved hypothetical protein.
Os06g0265100	4.51	4.43	**3.42**	Conserved hypothetical protein.
Os05g0247800	3.26	1.36	**3.25**	Glycoside hydrolase, family 18 protein.
Os03g0299700	4.93	5.52	**3.22**	Conserved hypothetical protein.
Os04g0398000	2.21	1.15	**2.87**	Pathogenesis-related transcriptional factor and ERF domain containing.
Os07g0162450	4.96	5.48	**2.83**	Conserved hypothetical protein.
Os11g0700900	2.41	1.34	**2.73**	Conserved hypothetical protein.
Os06g0725000	1.90	1.22	**2.63**	Similar to Ntdin.
Os05g0542500	1.81	1.26	**2.44**	LEA-like protein.
Os02g0441000	1.16	2.24	**2.40**	Conserved hypothetical protein.
Os02g0513100	2.02	2.04	**1.86**	Similar to MtN3 protein precursor.
Os03g0826900	2.73	2.41	**1.52**	Conserved hypothetical protein.
Os10g0378500	3.12	1.58	**1.34**	Conserved hypothetical protein.
Os08g0246800	1.09	1.09	**1.27**	Conserved hypothetical protein.
Os11g0451700	2.41	1.75	**1.27**	Similar to Dehydrin DHN1 (M3) (RAB-17 protein).
Os07g0511400	1.03	1.05	**1.21**	Hypothetical protein.
Os08g0193600	1.73	1.23	**1.19**	Cyclin-like F-box domain containing protein.
Os03g0286900	2.53	1.05	0.84	Similar to Low-temperature induced protein lt101.2.
Os03g0604500	1.68	1.18	0.73	Conserved hypothetical protein.
Os06g0662550	2.45	1.05	0.67	Conserved hypothetical protein.
Os11g0518900	2.92	1.28	0.59	Conserved hypothetical protein.
Os02g0252400	3.58	1.89	0.55	Similar to Zn finger protein (Fragment).
U60097	2.18	1.51	0.48	NA
Os10g0567900	2.57	3.22	0.41	HAT dimerisation domain containing protein.
Os08g0508000	3.27	1.31	0.35	Cytochrome P450 family protein.
Os12g0257800	2.54	1.16	0.30	Similar to Laccase (Fragment).
Os02g0168200	3.51	1.20	0.01	Similar to Transfactor-like protein.
Os07g0475900	1.74	1.02	-0.03	Amino acid-binding ACT domain containing protein.
Os03g0141100	1.72	3.02	-0.05	Conserved hypothetical protein.
Os02g0161700	3.72	1.93	-0.24	Leucine rich repeat, N-terminal domain containing protein.
Os11g0471200	1.98	2.95	-0.38	Conserved hypothetical protein.
Os02g0216200	2.19	3.63	-0.42	Hypothetical protein.
Os03g0423300	2.34	1.17	-0.44	Similar to Stearoyl-acyl carrier protein desaturse.
Os03g0693700	4.80	2.97	-0.49	Similar to Oxalate oxidase 1 (Germin).
Os04g0460300	1.28	1.05	-0.55	Amino acid/polyamine transporter II family protein.
Os12g0113500	2.07	1.17	-0.84	Similar to Protein kinase PK4.
Os01g0204900	1.66	1.58	-1.02	Conserved hypothetical protein.
Os06g0146800	3.12	1.75	-1.53	Conserved hypothetical protein.
Os03g0184550	2.72	1.08	-1.78	NAD-dependent epimerase/dehydratase family protein.
Os12g0438600	1.97	1.23	-1.97	Similar to Chloride channel protein CLC-a (AtCLC-a).
Os09g0294000	1.73	1.14	-2.06	Bifunctional aspartokinase/homoserine dehydrogenase 2
Os01g0860601	2.03	1.63	-2.10	Similar to Ferredoxin, root R-B1.
Os01g0734000	2.78	1.09	-2.12	Similar to WRKY DNA binding protein.
Os05g0369900	1.11	1.17	-2.76	Conserved hypothetical protein.
Os03g0386800	2.01	1.06	-3.35	Peptidase S10, serine carboxypeptidase family protein.
Os11g0655900	2.43	1.42	-3.54	Glutaredoxin-like, plant II family protein.
Os11g0559600	2.78	2.03	-4.02	Hypothetical protein.
Os01g0368900	2.48	1.75	-5.30	Similar to GLUTAREDOXIN.
Os09g0484900	2.91	1.04	-6.11	Sodium/sulphate symporter family protein.

Values are log2 sample/reference intensity ratios (P value < 0.05).
